# Longitudinal patterns of natural hazard exposures and anxiety and depression symptoms among young adults in four low- and middle-income countries

**DOI:** 10.1038/s41598-024-60106-6

**Published:** 2024-05-08

**Authors:** Ilan Cerna-Turoff, Joan A. Casey, Katherine Keyes, Kara E. Rudolph, Daniel Malinsky

**Affiliations:** 1 Mailman School of Public Health, Department of Environmental Health Sciences, Columbia University, New York City, NY USA; 2 School of Public Health, Department of Environmental and Occupational Health Sciences, University of Washington, Seattle, WA USA; 3 Mailman School of Public Health, Department of Epidemiology, Columbia University, New York City, NY USA; 4 Mailman School of Public Health, Department of Biostatistics, Columbia University, New York City, NY USA

**Keywords:** Psychology and behaviour, Epidemiology

## Abstract

We estimated the effect of community-level natural hazard exposure during prior developmental stages on later anxiety and depression symptoms among young adults and potential differences stratified by gender. We analyzed longitudinal data (2002–2020) on 5585 young adults between 19 and 26 years in Ethiopia, India, Peru, and Vietnam. A binary question identified community-level exposure, and psychometrically validated scales measured recent anxiety and depression symptoms. Young adults with three exposure histories (“time point 1,” “time point 2,” and “both time points”) were contrasted with their unexposed peers. We applied a longitudinal targeted minimum loss-based estimator with an ensemble of machine learning algorithms for estimation. Young adults living in exposed communities did not exhibit substantially different anxiety or depression symptoms from their unexposed peers, except for young women in Ethiopia who exhibited less anxiety symptoms (average causal effect [ACE] estimate = − 8.86 [95% CI: − 17.04, − 0.68] anxiety score). In this study, singular and repeated natural hazard exposures generally were not associated with later anxiety and depression symptoms. Further examination is needed to understand how distal natural hazard exposures affect lifelong mental health, which aspects of natural hazards are most salient, how disaster relief may modify symptoms, and gendered, age-specific, and contextual differences.

## Introduction

Natural hazards, such as tropical cyclones and earthquakes, are disruptive events that adversely affect populations globally. Over the past 20 years, natural hazards increased in their number and severity, claiming an estimated 1.23 million lives and affecting over four billion people^1^. A sizeable percentage of the affected population exhibits negative mental health symptoms and psychopathology after exposure to these events^2^. Associations between recent exposure to natural hazards and measures of impaired mental health have been found after the Mount Saint Helens volcanic eruption in the United States^3^, the Canterbury earthquake in New Zealand^4^, and a super-cyclone in India^5^, among many other disasters. Younger individuals often exhibit worse mental health symptoms compared to adults^6^. Considering that natural hazards disproportionately affect low- and middle-income countries where the majority of the population is under the age of 30, a large segment of young people have the potential to experience negative mental health symptoms as a result of exposure^7,8^.

Natural hazards may negatively affect mental health among young adults by either giving rise to molecular changes that increase susceptibility to mental health conditions or by changing the socioecological environment^9–12^. Life course epidemiology posits that specific developmental periods and transition points are sensitive and therefore, can exert a greater influence on later health^13^. In particular, early childhood and adolescence are two critical stages of development^14^. From birth until adolescence, the brain undergoes a rapid period of development, and high levels of neuroplasticity increase susceptibility to environmental stressors^15^. The body responds to stress by producing a glucocorticoid, cortisol, which can pass through the blood–brain barrier to shape neural development. As outlined by Tottenham and Sheridan^16^, an extensive body of animal and human studies document how cortisol alters the size, complexity, and activity of receptors in the brain that control socioemotional functioning. Stress-related brain structures develop at different stages, plateauing after the age of 25 years, so environmental exposures at several points in childhood, adolescence, and young adulthood are likely to be important for mental health over the life course^16^. Studies of hurricanes^9^ and earthquakes^10,11^ have found that children and adults experience changes in brain function and structure after exposure, which relate to stress and emotional reactivity in young adulthood and later life.

Social epidemiology proposes that natural hazards alter the context where a child lives and grows. Changes to the socioecological environment may lead to ongoing disadvantage that engenders the development of poor mental health in young adulthood^12^. After natural hazards, children may experience long-term separation from family members, displacement to new communities, or familial strain resulting from economic losses of their home or livelihood. Hurricane Katrina, for instance, produced separation of household heads and adult children at 2.2 and 2.7 times the national rate in a one-year period after exposure^17^. Amongst low-income parents, those who were relocated to new communities and those who were unstably housed had more psychological distress and perceived stress than returned households^18^. New or exacerbated economic vulnerability places children and adolescents at increased risk of child labor^19^, early marriage^20^, and other negative circumstances that can worsen their mental health and general wellbeing. Individual and household level vulnerability is further influenced by the overall economic context. Rubens et al.^21^ found that natural hazards were more strongly correlated with internalizing and externalizing among young people in countries with a medium Human Development Index (HDI) ($r$ = 0.56) versus high HDI ($r$ = 0.15)^21^. Regardless of context, gender has consistently been identified as an important factor, as girls tend to have worse mental health symptoms following natural hazard exposure than boys^6,21–23^.

Natural hazards are inherently multidimensional in that they have geophysical, atmospheric, hydrological, or other drivers that lead to multiple traumatic experiences during a disaster event. Spatial variability may also occur, even within small geographic areas. A lack of consensus exists in disaster studies regarding how to measure exposure. In practice, exposure has been operationalized as a series of questions of individual traumatic experiences to form scales, a continuous metric of area-level severity based upon aerial or sensor-based measures, a binary measure of exposure within administrative areas were the natural hazard occurred, or a binary measure of self-reported or community exposure^24^. Binary exposure measures on the community level are commonly used in low- and middle-income countries and serve as the exposure metric for this study^25–30^. Community-level exposures are then assigned to individuals. The socioecological model has been evoked as the theoretical basis for the validity of this exposure assignment, which posits that natural hazards change one’s environment and by way of this change, impact individuals^12,31^.

The current evidence excludes two important aspects of longitudinal exposures. One is that symptoms of poor mental health are often detected years after natural disaster exposure^32–36^. It warrants probing if distal exposures yield persistent negative effects. Second, young people increasingly are exposed to more than one natural hazard before reaching adulthood^37^. Natural hazards are common in low- and middle-income countries, but surprisingly, little is known about if repeat exposure affects mental health. This study aims to address these gaps by estimating how three patterns of natural hazard exposure histories influence later anxiety and depression symptoms among young adults in four low- and middle-income countries. Given the importance of gender in mental health symptoms, we further seek to estimate how exposure patterns may differ among young women and men.

## Results

**Figure 1:**
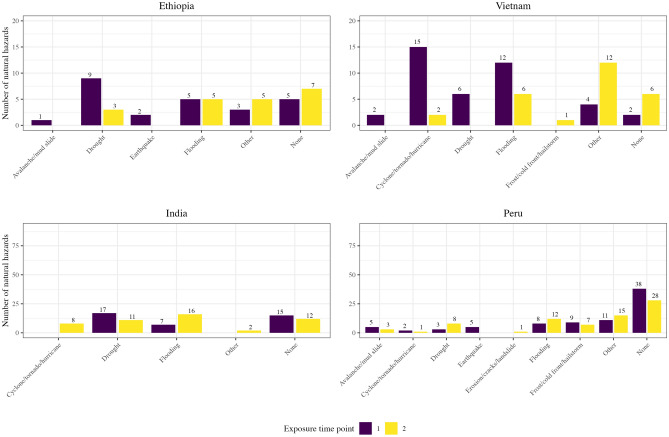
Number of natural hazards reported by community respondents at each exposure time point across communities. Summarized as counts and color coded by the first exposure time point (participants were under eight years of age) and second exposure time point (participants were 15 and 22 years, respectively). Exposure to natural hazards was measured at the community level. The same disaster event could be reported multiple times by different community respondents, so these values should be interpreted as general descriptive trends. *Flooding* category includes floods from any cause, including flash floods and overflowing of lakes, seas, or rivers. *Erosion/cracks/landslides* were specified as not resulting from earthquakes or other listed natural hazards.

A total of 5585 young adults were included in the final analysis (Ethiopia: 1766, India: 844, Peru: 1232, and Vietnam: 1743) who lived in 158 communities (Ethiopia: 19, India: 39, Peru: 75, and Vietnam: 25). The distribution of covariates remained similar before and after imputation of the missing data (see Supplementary Tab. S1). After excluding communities for practical positivity violations, the most commonly reported natural hazard types were drought for Ethiopia (36.4%) and India (45.9%); unspecified types of natural hazards in Peru (28.9%); and flooding in Vietnam (30.0%) (Fig. 1). A natural hazard occurred in most young adult’s communities (68.3%) at the measured time points. Comparing across countries, the percentages ranged from 57.7% exposed in Peru to 88.2% exposed in Vietnam. Communities were likewise frequently exposed to more than one natural hazard, with Vietnam having the highest average of 2.5 natural hazards per community.

Most GAD-7 and PHQ-8 scores were low in the final sample used for analysis (Table 1), with exception of Peru which had average and median scores that were in the mild range for anxiety and depression symptoms (see Supplementary Fig. S1).

**Table 1:** Distribution of GAD-7 and PHQ-8 scores among young adults in the 2020 telephone survey.

	GAD-7					PHQ-8				
	Mean (SD)	Median	75th percentile	Min	Max	Mean (SD)	Median	75th percentile	Min	Max
Ethiopia	2.14 (3.06)	1.00	4.00	0.00	19.00	1.93 (2.80)	0.00	3.00	0.00	21.00
India	1.35 (2.04)	0.00	2.00	0.00	14.00	1.13 (1.95)	0.00	2.00	0.00	13.00
Peru	4.46 (4.18)	3.00	7.00	0.00	19.00	3.52 (3.78)	2.00	5.00	0.00	22.00
Vietnam	1.52 (2.51)	0.00	2.00	0.00	18.00	1.26 (2.37)	0.00	2.00	0.00	17.00

SD = standard deviation; Min = minimum; Max = maximum; GAD-7 = Generalized Anxiety Disorder scale-7; PHQ-8 = Patient Health Questionnaire depression scale-8. Total sample size = 5585. Young adults were aged 19 and 26 years in 2020.

Table 2 presents the average differences between young adults with each exposure pattern as compared to young adults who lived in communities that were never exposed. In the full sample, no pattern of exposure was associated with increases in anxiety and depression symptoms. Some estimates (e.g., repeat exposure in Vietnam) exhibited wide confidence intervals, which illustrates substantial uncertainty in the effect estimates for these exposure patterns in this dataset.

**Table 2:** Estimated effect of natural hazard exposure patterns on anxiety and depression scores.

Exposure	Ethiopia			India			Peru			Vietnam		
	Estimate	95% CI	*p*-value	Estimate	95% CI	*p*-value	Estimate	95% CI	*p*-value	Estimate	95% CI	*p*-value
GAD-7 scores												
Time point 1	− 0.57	− 3.22, 2.08	0.674	0.15	− 0.86, 0.56	0.681	0.42	− 1.50, 2.34	0.671	− 0.02	− 3.88, 3.85	0.994
Time point 2	− 0.94	− 2.84, 0.96	0.334	− 0.08	− 0.39, 0.22	0.593	− 2.27	− 5.42, 0.88	0.159	− 1.08	− 2.35, 0.18	0.093
Both time points	− 8.26	− 16.75, 0.24	0.057	− 0.11	− 0.86, 0.64	0.779	0.40	− 1.43, 2.23	0.668	1.22	− 2.75, 5.19	0.547
PHQ-8 scores												
Time point 1	− 0.57	− 4.74, 3.60	0.789	− 0.20	− 0.83, 0.44	0.544	0.38	− 1.49, 2.25	0.692	− 0.34	− 3.91, 3.23	0.851
Time point 2	− 0.87	− 2.39, 0.64	0.259	− 0.14	− 0.35, 0.07	0.204	− 1.82	− 6.17, 2.52	0.410	− 0.93	− 6.46, 4.60	0.742
Both time points	− 3.15	− 13.82, 7.52	0.563	− 0.25	− 1.15, 0.66	0.592	0.21	− 1.42, 1.85	0.796	1.17	− 1.85, 4.20	0.447

CI = confidence intervals; GAD-7 = Generalized Anxiety Disorder scale-7; PHQ-8 = Patient Health Questionnaire depression scale-8; Estimates and confidence intervals are rounded to two decimal places and *p*-values to three decimal places. The full list of individual-, household-, and community-level covariates included in each model are found in Supplementary Table S5.

When examining young men and women separately, a more nuanced pattern of mental health emerges. Ethiopian young women who lived in communities that were repeatedly exposed to natural hazards had GAD-7 scores that were 8.86 points lower [95% CI: -17.04, -0.68] than young women who had never been exposed (*p*-value = 0.034). A difference in PHQ-8 and GAD-7 scores were not identified in any other country or gender subgroup (Table 3).

**Table 3:** Subgroup analysis by gender of the estimated effect of natural hazard exposure patterns on anxiety and depression scores.

Exposure	Ethiopia			India			Peru			Vietnam		
	Estimate	95% CI	*p*-value	Estimate	95% CI	*p*-value	Estimate	95% CI	*p*-value	Estimate	95% CI	*p*-value
Girls—GAD-7 scores												
Time point 1	− 1.84	− 4.27, 0.59	0.137	− 0.41	− 1.74, 0.92	0.544	1.23	− 1.12, 3.58	0.306	− 0.48	− 4.75, 3.79	0.825
Time point 2	− 1.73	− 3.78, 0.32	0.100	− 0.24	− 0.58, 0.11	0.176	0.15	− 0.95, 1.24	0.795	− 1.20	− 22.99, 20.60	0.914
Both time points	**− 8.86**	**− 17.04, − 0.68**	**0.034**	0.19	− 1.07, 1.45	0.768	0.59	− 1.53, 2.72	0.584	1.34	− 2.26, 4.94	0.465
Girls—PHQ-8 scores												
Time point 1	− 1.02	− 3.53, 1.49	0.427	− 0.06	− 1.07, 0.95	0.907	0.57	− 1.25, 2.40	0.539	− 0.41	− 3.61, 2.80	0.803
Time point 2	− 0.82	− 2.79, 1.14	0.412	− 0.23	− 0.47, 0.02	0.071	− 0.35	− 1.47, 0.78	0.543	− 1.92	− 9.92, 6.08	0.638
Both time points	− 7.63	− 19.44, 4.19	0.206	− 0.35	− 1.60, 0.90	0.586	0.45	− 1.31, 2.20	0.619	1.47	− 0.83, 3.78	0.211

Exposure	Ethiopia			India			Peru			Vietnam		
	Estimate	95% CI	*p*-value	Estimate	95% CI	*p*-value	Estimate	95% CI	*p*-value	Estimate	95% CI	*p*-value
Boys—GAD-7 scores												
Time point 1	− 0.25	− 2.84, 2.33	0.848	− 0.02	− 1.01, 0.98	0.975	2.68	− 1.67, 7.03	0.227	0.68	− 1.52, 2.88	0.545
Time point 2	− 0.93	− 2.99, 1.12	0.372	0.00	− 0.14, 0.15	0.960	2.21	− 1.66, 6.07	0.263	0.70	− 2.85, 4.25	0.697
Both time points	− 7.16	− 16.36, 2.05	0.128	− 0.10	− 0.85, 0.65	0.785	2.82	− 1.32, 6.96	0.182	− 0.11	− 3.39, 3.18	0.950
Boys—PHQ-8 scores												
Time point 1	0.40	− 2.44, 3.24	0.781	− 0.39	− 1.13, 0.34	0.296	2.55	− 2.51, 7.61	0.324	0.13	− 1.68, 1.94	0.887
Time point 2	− 0.41	− 2.02, 1.21	0.623	0.00	− 0.31, 0.32	0.981	1.75	− 3.03, 6.53	0.473	0.76	− 4.89, 6.42	0.791
Both time points	− 4.99	− 19.68, 9.71	0.506	− 0.21	− 1.03, 0.60	0.611	2.60	− 2.55, 7.74	0.322	− 0.29	− 2.29, 1.71	0.775

CI = confidence intervals; GAD-7 = Generalized Anxiety Disorder scale-7; PHQ-8 = Patient Health Questionnaire depression scale-8. Estimates and confidence intervals are rounded to two decimal places and *p*-values to three decimal places. Significant estimates under *p*-value < 0.05 are indicated in bold.

## Discussion

Young adults who lived in communities that were exposed to any natural hazard exposure pattern did not have substantially different GAD-7 and PHQ-8 scores from their unexposed peers. Prior exposure was not associated with increased risk of later anxiety and depression symptoms in young adulthood in these four countries, regardless of context, and effects were not magnified when young people were exposed at both time points. Past studies have found mixed associations between exposure to natural hazards and mental health, likely due to differences in exposure measurement, study population, and study design. A global meta-analysis contrasting disaster exposed to unexposed groups and incorporating pre-post testing found slight increases in depression among the exposed population (standardized mean difference [SMD] = 0.55, 95% CI: 0.05, 1.06). Anxiety symptoms, however, did not conclusively differ^38^. Specific to children, Norris et al.^6^ found mixed but generally worse mental health symptoms among younger age groups as compared to adults across multiple studies. Another meta-analytic study similarly estimated a slight increase in combined anxiety and depression metrics after exposure for youth ($r$ = 0.18, 95% CI: 0.14, 0.22)^21^, and a recent systematic review of post-disaster mental health recovery from 29 countries found that depression remained elevated for years after natural disaster or pandemic exposure among young people and at a higher rate than in adult populations^39^. Amongst these studies, exposure was commonly measured in low- and middle-income countries as residence in a geographic area affected by the natural hazard (e.g.,^25–30^). The current literature tends to indicate that exposure to natural hazards induces a slight but lasting increase in depression and anxiety symptoms among young people, with additional variation depending on the severity, duration, and personal impact of the natural hazard. In contrast, this study generally did not find evidence that exposure to natural hazards related to anxiety or depression symptoms. The findings support possible conclusions about the frequency and historical/longitudinal patterns of exposure (i.e., repeat exposure, exposure in adolescence/young adulthood, and exposures in early life) which are not typical of studies that examine one natural hazard event, and they complement existing evidence involving “dose” of exposure from proximity or severity to natural hazards.

The type and severity of traumatic experiences over one’s lifetime and during the natural hazard may be most predictive in determining who experiences poor mental health. Tang et al.^40^ found that across studies of children, depression symptoms only significantly increased among subgroups that had prior experiences of trauma or specific negative experiences during and after the natural hazard (i.e., being trapped during the disaster; experiencing injury, fear, or bereavement during the disaster; witnessing injury or death; or having poor social support). Personal behaviors and worldviews additionally act to protect or exacerbate symptoms. Stoicism and maladaptive coping, such as venting and distraction, can lead to greater deterioration of mental health, whereas acceptance, positive reframing, and humor are protective^41^. Similarly, individuals that use religious coping to find meaning and positive lessons in negative events often have overall better mental health^42^. Young people, likewise, are embedded within families, communities, and societies that influence their mental health symptoms and recovery. Familial and social support as well as the overall sociopolitical context influence the development of symptoms of poor mental health after natural hazards^21,43,44^. This nuance collectively points to a complicated pattern of individual and socioecological factors that may modify the relationship between natural hazards and mental health. Although our study sought to adjust for all meaningful pre-exposure covariates in the dataset, individual, relational, and societal dimensions of mental health response are not fully understood or sufficiently captured in data on disaster-related mental health. Furthermore, a lack of consensus exists on how to best operationalize exposure, and measures of exposure at the community or individual level may differ in their sensitivity. Exposure has been constructed using individual disaster-related stressors or area-level measures of proximity, severity, and general residence in an affected area^24^. These measurements merit further exploration among young people in future research.

Unexpectedly, young women in Ethiopia exhibited lower GAD-7 scores (less reported anxiety) when living in communities that were repeatedly exposed to natural hazards at both time points. The literature largely finds that women have worse mental health symptomology than men when exposed to natural hazards^6,21–23^. Ethiopia is likely no exception. Of the limited evidence, a cross-sectional study from the capital of Addis Ababa found that woman had 1.74 (95% CI: 1.21, 2.50) higher adjusted odds of posttraumatic stress disorder (PTSD) after a landslide^45^. School-based samples among adolescents and young adults from various parts of the country further indicate that female gender is associated with higher depression and anxiety symptoms^46–48^. The lower anxiety symptoms among repeatedly exposed young women in Ethiopia may be an artifact of unmeasured confounding from factors that were not included in this study. One such covariate would be the structure of disaster relief aid. It is plausible that a greater amount of gender-sensitive aid was allocated to communities where natural hazards repeatedly occurred, as compared to communities that did not experience natural hazards. Ethiopia receives some of the world’s highest amount of foreign aid and hosts several national safety net programs that provide gender-responsive support to households affected by drought and other climate-related shocks^49,50^. It may be that young women in affected communities benefited from this investment in early childhood disproportionate to their peers and therefore, had lower anxiety for their future wellbeing when repeated natural hazards occurred. When baseline services are not well-distributed nationally, and one group receives targeted aid, asymmetries commonly occur^51^. Ethiopia has had mixed success in its aid distribution and gender-responsiveness^52,53^; however, qualitative evidence collected as part of a longitudinal study with Ethiopian adolescents and young adults, aged 10 to 20 years, corroborates that climate mitigation strategies led to investment in gender-sensitive social protection programs, which positively impacted young women and girls^50^. Young women who lived in communities where these programs were implemented may have benefited over the long-term. Furthermore, the study measured anxiety and depression during the height of the COVID-19 pandemic. Poor households in rural areas were 88.0% more likely to receive government assistance within the 10 months after the start of the pandemic than non-poor households. These households were disproportionately female headed and overlapped with areas of the country where natural hazards frequently occur^54^. COVID-19 investment could have lowered recent anxiety symptoms among young women in these communities. One’s perception of the government’s response to natural hazards may be as important as the actual aid received in influencing mental health symptoms. Although a different context, an estimated 18.0% of survivors of the Southeast Asian earthquake and tsunami in Thailand newly developed posttraumatic stress symptoms when they perceived that there was a low level of government support^55^.

We applied a doubly robust estimator of longitudinal effects to estimate the extent to which different patterns of natural hazard exposure affected later anxiety and depression symptoms. In addition to its doubly robust property (i.e., it produces an unbiased estimate if either the model for the exposure or the model for the outcome is consistent), this estimator allows for the incorporation of machine learning algorithms in model fitting while retaining theory-based inference and appropriately accounts for time-varying confounders affected by prior exposure^56^. It further is semi-parametrically efficient if both exposure and outcome models are consistent^57^. Parametric models, like standard regression, suffer from substantial bias if the functional form is misspecified (e.g., a linear form is incorrectly assumed for how covariates relate to the outcome). Nonparametric approaches, such as the one implemented in this analysis, have been shown to be less biased and more robust for estimating causal effects^58^. The use of machine learning ensembles to estimate these “nuisance” models further avoids reliance on potentially strong parametric assumptions and has as high or higher accuracy than individual models^59,60^. The study employed psychometrically validated mental health scales that have been widely used with young adults in a variety of cultural settings^61–66^. Depression and general anxiety are the two most common mental health symptoms and are common after natural hazard exposure^40,67^. Extensive research has documented the connection between singular exposure to natural hazards and poor mental health^2–4^ and identified young people as vulnerable to poor mental health shortly after exposure^6,21,23^. The majority of studies, however, originate from high-income countries, use cross-sectional designs, and focus on a singular and recent event^2–4^. This study builds on each of these respective areas of the literature. In particular, the adoption of a life course approach that seeks to quantify long-term mental health consequences of natural hazard exposures is a distinct framing in the disaster literature. Our results did not provide a clear pattern, and so, further research should consider collection of longitudinal information on natural hazard exposure over several critical developmental stages.

Our study also has several limitations. Community respondents reported on community-level exposure to natural hazards, which does not fully capture individual experiences of young adults during disaster events or discordance between individuals and community members in their reporting. Certain types of natural hazards may affect a segment of the community, leading to differential exposure to the natural hazard within the same area (e.g., the difference between living on hilltops versus valleys when community flooding occurs). This analysis should therefore be interpreted as estimating differences in the average mental health of young adults living in communities that experienced and did not experience natural hazards. Substantial debate exists as to the best way to measure exposure to natural hazards, but it is widely acknowledged that living in communities affected by natural hazard will disrupt the socioecological environment to some extent, regardless of the severity of personal experiences, and community exposure is deemed a valid metric in the disaster literature^24,68^. Relatedly, information on individual experiences during natural hazards was not captured in the Young Lives study. Specific experiences—namely, damage to one’s house and belongings, perceived or experienced danger, and illness or injury to one’s self or others—in validated trauma scales are most indicative of poor mental after natural hazard exposure^69,70^. We did not have access to validated scales to contrast individual experiences, but we hypothesize that it would have been more sensitive than binary reporting of community exposures. Alternative metrics for measuring exposure on a continuous scale, such as strength of, proximity to, or damages from a natural hazard, would capture how specific levels or severity of exposure influenced mental health outcomes. It may be that mental health worsens as proximity and impact of exposure increases. Nevertheless, binary measures of total community exposure can estimate the overall effect in the target population^24^. Furthermore, recall bias may exist in exposure reporting. Although community respondents are likely to remember major natural hazards, they may have underreported minor natural hazards, which would artificially make the exposed and unexposed groups seem more similar than they were in actuality and bias towards the null. In addition, this dataset did not consistently collect information on exposure types at each wave, which precluded analysis of the influence of specific types of natural hazards or the creation of a count for the number of natural hazards that occurred in a community. It may also be that other developmental periods are more influential on mental health in young adulthood. The two time periods used in this study, however, are emphasized in global health strategies for intervening on lifelong health^14^, and other measures were not comparable. Last, we were interested in distal exposures that happened in early childhood and/or four years prior to the outcome measurement. For the majority of individuals who exhibit poor mental health after natural hazard exposure, symptoms are short lived and improve over time^6,21,23^. It may be that effects would have been detected if we utilized a shorter time frame between exposure and outcome. Nevertheless, a segment of the population continues to experience symptoms of poor mental health years after natural disaster exposure^32–36^. A recent multilingual systematic review found that specifically anxiety and depression symptoms remain elevated for years after exposure to disasters and pandemics and that young people having higher rates than adult populations (*p* < 0.005)^39^. It, therefore, merits understanding if patterns can be detected in later developmental stages.

In terms of outcome measures, anxiety and depression symptoms were not measured in earlier data collection. We adjusted for a measure of past subjective wellbeing, but subjective wellbeing does not completely overlap with anxiety and depression constructs. Although widely used, standardized scales do not capture culturally specific expressions of anxiety and depression. Poor mental health is commonly expressed as somatization in non-Western societies. For instance, the Luo of South Sudan define depression as “*nger yec*,” a conglomeration of symptoms that overlap with Western definitions of depression but also include stomach pain and diarrhea^71^. Similar somatic symptoms have been found among Ethiopian^72^, Indian^73^, Peruvian^74^, and Vietnamese populations^75^. Last, these selected scales did not screen for other expressions of poor mental health. It may be that young adults had other mental health conditions, such as PTSD, which were outside of the purview of this study.

## Methods

This study adhered to STROBE guidelines for observational studies (see Supplementary Tab. S2)^76^. The Institutional Review Board at the University of Oxford as well as country-specific ethical review boards approved the initial data collection, and informed consent was obtained from all study participants^77^. This analysis was conducted on secondary data and deemed exempt from Columbia University’s Institutional Review Board (IRB-AAAT9321).

## Sample and study design

The Young Lives study followed a cohort of children from birth to young adulthood in Ethiopia, India, Peru, and Vietnam from 2002 to the present^78^. Five waves of data collection occurred at regular intervals every three to four years from 2002 to 2016. Sentinel sites were selected from poorer regions of each country using a three-stage sampling frame^77^. The primary data collection consisted of multiple linked surveys at each time point. Our study merged information from surveys administered to adult caregivers, community respondents, and young adults (Table 4).

**Table 4:** Sources of information for study design.

	Adult caregivers	Community respondents	Young adults
Study component	1. Participant-level covariates	1. Community-level covariates 2. Exposures	1. Participant-level covariates 2. Outcomes
Data source	Household survey	Community leader/informant survey	Household survey Telephone survey

The young adults were recruited as children as well as their adult caregivers. Within sentinel sites, households containing children within the target age group were randomly selected^77^. Refusal rates were low at baseline (under 2.0%), and replacement sampling was used to obtain a sufficient number of participants^79^. The initial cohort was gender balanced and targeted 3000 children for each country (2000 children between six and eighteen months and 1000 children between seven to eight years). Within each household, an adult caregiver provided information on the selected child at baseline and subsequent waves into early childhood. As children, young adults began to self-report upon reaching the age of eight years and increasingly provided information on themselves and their households into young adulthood. In addition, community respondents reported on natural hazard exposures in the locality where the participants lived and characteristics of those communities at each exposure time point. A purposeful sample of community respondents was selected at each wave by national field team supervisors. Community respondents included teachers, government officials, and other local leaders^77^. The sixth wave of data collection was adapted for the COVID-19 pandemic. In 2020, a series of three rounds of telephone surveys were conducted, during which young adults self-reported on their recent anxiety and depression symptoms. The participants were young adults in 2020, aged 19 and 26 years. Of the original sample of 11,784 children, 83.7% of the participants were retained across survey waves and into the 2020 telephone surveys^80^.

## Definition of exposure

Community respondents were asked a series of binary questions on exposure to different natural hazards at each survey wave between 2002 until 2016 (*“In the past [number of years since last survey round], has any of the following natural disasters occurred in this locality?”*). The original baseline survey in 2002 asked community respondents about natural disasters in the past two years. We organized the responses into ten non-overlapping categories of geophysical and climactic natural hazards and a category of no exposure (see Supplementary Tab. S3). Due to inconsistent documentation of natural hazard types at each survey wave and the potential for duplicate reporting of the same disaster event by different community respondents, we aggregated exposure across types to create a binary exposure (i.e., a community was exposed if one or more natural hazards were reported and unexposed if no natural hazard was reported). Each young adult was then assigned a community exposure based upon consistently living in a community since the last survey wave. We further restricted exposure to two survey waves that were meaningful stages in child development and collected consistent exposure information—the first survey wave in 2002 and the fifth survey wave in 2016. Our study design therefore had three possible exposure regimes: (a) “time point 1”: exposure to one or more natural hazards in 2002 when participants were under eight years of age; (b) “time point 2”: exposure to one or more natural hazards in 2016 when participants were 15 and 22 years old, respectively; and (c) “both time points”: exposure to one or more natural hazards in 2002 and 2016.

Expected mental health scores were estimated for participants under each of these potential regimes and contrasted to a regime in which individuals lived in communities that were never exposed to natural hazards. The causal contrasts of interest were the expected differences in mental health scores under each exposure regime versus never being exposed. These estimates may be interpreted as average causal effects (ACEs) under the assumption that there were no unmeasured sources of confounding (see Supplementary Tab. S4)^56^.

## Definition of outcomes

Young adults reported their recent anxiety and depression symptoms in two of the three rounds of telephone surveys in 2020, using psychometrically validated scales—the Generalized Anxiety Disorder-7 (GAD-7) and the Patient Health Questionnaire depression-8 (PHQ-8). The PHQ-8 is an eight-question screener that measures depressive incidents in the past two weeks^81^, and the GAD-7 is a seven-item questionnaire that measures symptoms of anxiety in the past two weeks^82^. Both tools have a strong overlap with the Diagnostic and Statistical Manual of Mental Disorders (DSM-IV) criteria^81,82^. They have been extensively used globally with young adults and have been validated in the four study countries^83–86^. GAD-7 and PHQ-8 scores were collected twice; we sought to avoid possible biases from regression towards the mean often exhibited in repeat psychological testing and seasonal differences in mental health symptoms by analyzing the first time that mental health scores were recorded^87^. Only participants who answered all questions in the series were included in the analysis to maintain the psychometric validity of the mental health scales (percentage of participants missing items for GAD-7 and PHQ-8 scales: less than 1.0% for Ethiopia, India, and Vietnam and approximately 1.7% for Peru).

## Covariates

Based on epidemiological theory about the correlates of mental health outcomes and factors related to natural hazard exposure, we included a wide selection of individual-, household-, and community-level covariates (see Supplementary Tab. S5). Additional information on data preparation can be found in previous work^88^. Gender was a key covariate included in the outcome models to reflect that women tend to have worse mental health symptoms after exposure to disaster events^89,90^. Covariates varied in their percentage of missingness from less than 1.0% to 36.3%. For all missing covariates, we conducted multiple imputation to analyze the dataset as if it was complete, in line with past recommendations^91^.

## Analyses

We applied a longitudinal targeted minimum loss-based estimator to estimate anxiety and depression scores under exposure regimes, using the lmtp package in the R statistical software^92,93^. This estimator is doubly robust and non-parametric^56^. We fit the models for treatment and outcome using an ensemble of machine learning algorithms in SuperLearner^59^. The ensemble included the sample mean, generalized linear models, tree-based algorithms (extreme gradient boosting), and spline methods (multivariate adaptive regression splines and general additive models) and used five-fold cross-validation to select an optimal combination. We further added trimming at the 99^th^ percentile to exclude extreme weights in estimation. An analysis stratified by gender was conducted to identify differential effects. 

The lmtp package allows for specification of covariates separately in exposure, censoring, and outcome models to mitigate practical positivity violations^94^. To reduce the extent of practical positivity violations, we restricted analyses to geographic areas where the risk of natural hazards was more varied, conditional on covariates (Ethiopia: excluded desert communities [29.9%], India: excluded hill communities and regions that consisted only of inland plains [67.4%], and Vietnam: excluded the Red River region [22.9%]). In the excluded areas, the participants were always exposed to natural hazards (with no “unexposed” comparison peers), and therefore, these regions violated the positivity identification assumption. We also restricted the data to those individuals who did not move to different communities between the first and second exposure time points to reduce potential exposure misclassification. We incorporated community clustering for standard error calculation. 

## Conclusions

Climate change will continue to spur increases in the frequency and severity natural hazards. Globally, a majority of young people live in low- and middle-income countries that are disproportionately affected by climate change^7,8^. It is essential to better understand the effect of distal natural hazards exposures on later mental health. While encouraging that we did not observe increased anxiety and depression symptoms among exposed individuals, information gaps exist as to which developmental period and the number and type of natural hazard exposure that might lead to future poor mental health. Comprehensive and consistent collection of data on location, timing, and intensity of exposures is needed to ensure that we accurately pinpoint health effects. Great care must be taken in future study design to collect disaggregated information on recipients by age and gender and levels/types of aid. The call for better documentation should be integrated into disaster response and climate change policies, and high-quality evidence should drive structures for mental health service provision among the most impacted populations.

### Supplementary Information

Supplementary Information.

## Data Availability

The underlying data used in this analysis can be accessed from the UK Data Service, a public social science digital repository (https://beta.ukdataservice.ac.uk/datacatalogue/series/series?id=2000060).

## Code availability

The R scripts that accompany this analysis can be found on GitHub (https://github.com/ilan-cerna-turoff/lmtp-hazards-mental-health.git).

## References

[1] UCLouvain, USAID & Centre for research on the epidemiology of disasters. Human cost of disasters (2000–2019). *Centre for research on the epidemiology of disasters*. https://www.undrr.org/publication/human-cost-disasters-overview-last-20-years-2000-2019#:~:text=In%20the%20period%202000%20to,over%20the%20previous%20twenty%20years (2020).

[2] Sharpe I, Davison CM (2021). Climate change, climate-related disasters and mental disorder in low- And middle-income countries: a scoping review. BMJ Open.

[3] Adams PR, Adams GR (1984). Mount Saint Helens’ ashfall: evidence for a disaster stress reaction. Am. Psychol..

[4] Beaglehole B, Mulder RT, Boden JM, Bell CJ (2019). A systematic review of the psychological impacts of the Canterbury earthquakes on mental health. Aust. N. Z. J. Public Health.

[5] Kar N (2007). Post-traumatic stress disorder in children and adolescents one year after a super-cyclone in Orissa, India: Exploring cross-cultural validity and vulnerability factors. BMC Psychiatry.

[6] Norris FH (2002). 60,000 disaster victims speak: part I: An empirical review of the empirical literature, 1981–2001. Psychiatry J..

[7] Peduzzi P, Dao H, Herold C, Mouton F (2009). Assessing global exposure and vulnerability towards natural hazards: the Disaster Risk Index. Nat. Hazards Earth Syst. Sci..

[8] United Nations - Department of Economic and Social Affairs, Population Division. World population prospects 2019: volume II, demographic profiles, 2019 revision. ST/ESA/SER.A/427. *United Nations* https://www.un.org/development/desa/pd/news/world-population-prospects-2019-0 (2019).

[9] Kessel EM (2018). Hurricane Sandy exposure alters the development of neural reactivity to negative stimuli in children. Child Dev..

[10] Lui S (2009). High-field MRI reveals an acute impact on brain function in survivors of the magnitude 8.0 earthquake in China. Proc. Natl. Acad. Sci. U.S.A..

[11] Sekiguchi A (2013). Brain structural changes as vulnerability factors and acquired signs of post-earthquake stress. Mol. Psychiatry.

[12] Abramson DM, Stehling-Ariza T, Park YS, Walsh L, Culp D (2010). Measuring individual disaster recovery: a socioecological framework. Disaster Med. Public Health Prep..

[13] Kuh D (2003). Life course epidemiology. J. Epidemiol. Commun. Health.

[14] Bundy D. A. P. *et al.* Child and adolescent health and development: realizing neglected potential in *Child and adolescent health and development: realizing neglected potential* (eds. Bundy D. A. P. *et al.*) 1–23 (World Bank, 2017). 30212145

[15] Casey BJ, Giedd JN, Thomas KM (2000). Structural and functional brain development and its relation to cognitive development. Biol. Psychol..

[16] Tottenham N, Sheridan MA (2009). A review of adversity, the amygdala and the hippocampus: a consideration of developmental timing. Front Hum. Neurosci..

[17] Rendall MS (2011). Breakup of New Orleans households after Hurricane Katrina. J. Marriage Fam..

[18] Fussell E, Lowe SR (2014). The impact of housing displacement on the mental health of low-income parents after Hurricane Katrina. Soc. Sci. Med..

[19] Beegle K, Dehejia RH, Gatti R (2006). Child labor and agricultural shocks. J. Dev. Econ..

[20] KumalaDewi LPR, Dartanto T (2019). Natural disasters and girls vulnerability: is child marriage a coping strategy of economic shocks in Indonesia?. Vulnerable Child Youth Stud..

[21] Rubens SL, Felix ED, Hambrick EP (2018). A meta-analysis of the impact of natural disasters on internalizing and externalizing problems in youth. J. Trauma Stress.

[22] Tang W (2017). Mental health problems among children and adolescents experiencing two major earthquakes in remote mountainous regions: A longitudinal study. Compr. Psychiatry.

[23] Rezayat AA (2020). Evaluating the prevalence of PTSD among children and adolescents after earthquakes and floods: a systematic review and meta-analysis. Psychiatr. Q..

[24] Chan CS, Rhodes JE (2014). Measuring exposure in Hurricane Katrina: a meta-analysis and an integrative data analysis. PLoS One.

[25] Fan F (2017). Cohort profile: The Wenchuan earthquake adolescent health cohort study. Int. J. Epidemiol..

[26] Altindag A, Ozen S, Sir A (2005). One-year follow-up study of posttraumatic stress disorder among earthquake survivors in Turkey. Compr. Psychiatry.

[27] Berger R, Gelkopf M (2009). School-based intervention for the treatment of tsunami-related distress in children: a quasi randomized controlled trial. Psychother. Psychosom..

[28] Frankenberg E, Nobles J, Sumantri C (2012). Community destruction and traumatic stress in post-tsunami Indonesia. J. Health Soc. Behav..

[29] Chui CHK (2017). Predictive factors of depression symptoms among adolescents in the 18-month follow-up after Wenchuan earthquake in China. J. Ment. Health.

[30] Andrades M, García FE, Kilmer RP (2021). Post-traumatic stress symptoms and post-traumatic growth in children and adolescents 12 months and 24 months after the earthquake and tsunamis in Chile in 2010: a longitudinal study. Int. J. Psychol..

[31] Bronfenbrenner U (1977). Toward an experimental ecology of human development. Am. Psychol..

[32] McFarlane AC, Van Hooff M (2009). Impact of childhood exposure to a natural disaster on adult mental health: 20-Year longitudinal follow-up study. Br. J. Psychiatry.

[33] Ye Y, Fan F, Li L, Han Q (2014). Trajectory and predictors of depressive symptoms among adolescent survivors following the Wenchuan earthquake in China: A cohort study. Soc. Psychiatry Psychiatr. Epidemiol..

[34] Tian Y, Wong TKS, Li J, Jiang X (2014). Posttraumatic stress disorder and its risk factors among adolescent survivors three years after an 8.0 magnitude earthquake in China. BMC Public Health.

[35] Piyasil V (2011). Post-traumatic stress disorder in children after the tsunami disaster in Thailand: a 5-year follow-up. J. Med. Assoc. Thail..

[36] Bryant RA (2018). Longitudinal study of changing psychological outcomes following the Victorian Black Saturday bushfires. Aust. N. Z. J. Psychiatry.

[37] Thiery W (2021). Intergenerational inequities in exposure to climate extremes. Science.

[38] Beaglehole B (2018). Psychological distress and psychiatric disorder after natural disasters: systematic review and meta-analysis. Br. J. Psychiatry.

[39] Newnham EA (2022). Long term mental health trajectories after disasters and pandemics: a multilingual systematic review of prevalence, risk and protective factors. Clin. Psychol. Rev..

[40] Tang B, Liu X, Liu Y, Xue C, Zhang L (2014). A meta-analysis of risk factors for depression in adults and children after natural disasters. BMC Public Health.

[41] Bei B (2013). A prospective study of the impact of floods on the mental and physical health of older adults. Aging Ment. Health.

[42] Ano GG, Vasconcelles EB (2005). Religious coping and psychological adjustment to stress: A meta-analysis. J. Clin. Psychol..

[43] Wickrama KAS, Kaspar V (2007). Family context of mental health risk in tsunami-exposed adolescents: Findings from a pilot study in Sri Lanka. Soc. Sci. Med..

[44] Wind TR, Fordham M, Komproe IH (2011). Social capital and post-disaster mental health. Glob Health Action.

[45] Asnakew S, Shumet S, Ginbare W, Legas G, Haile K (2019). Prevalence of post-traumatic stress disorder and associated factors among Koshe landslide survivors, Addis Ababa, Ethiopia: a community-based, cross-sectional study. BMJ Open.

[46] Kebede MA, Anbessie B, Ayano G (2019). Prevalence and predictors of depression and anxiety among medical students in Addis Ababa Ethiopia. Int. J. Ment. Health Syst..

[47] Gebremedhin H (2020). Prevalence and associated factors of psychological distress among secondary school students in Mekelle City, Tigray Region, Ethiopia: cross-sectional study. Psychol. Res. Behav. Manag..

[48] Nakie G, Segon T, Melkam M, Desalegn GT, Zeleke TA (2022). Prevalence and associated factors of depression, anxiety, and stress among high school students in, Northwest Ethiopia, 2021. BMC Psychiatry.

[49] World Bank. Net official development assistance and official aid received (current US$) - Ethiopia. *Development Assistance Committee of the Organisation for Economic Co-operation and Development, Geographical Distribution of Financial Flows to Developing Countries, Development Co-operation Report, and International Development Statistics database* https://data.worldbank.org/indicator/DT.ODA.ALLD.CD (2023).

[50] Devonald M, Jones N, Iyasu GA, Yadete W (2022). Rethinking climate change through a gender and adolescent lens in Ethiopia. Clim. Dev..

[51] Herzer D. & Nunnenkamp P. The effect of foreign aid on income inequality: evidence from panel cointegration Standard-Nutzungsbedingungen. Kiel Working Paper No. 1762 *Leibniz Information Centre for Economics* http://hdl.handle.net/10419/56382 (2012).

[52] Jones N., Tafere Y. & Woldehanna T. Gendered risks, poverty and vulnerability in Ethiopia: to what extent is the Productive Safety Net Programme (PSNP) making a difference? *Overseas Development Institute* www.odi.org.uk (2010).

[53] Briggs RC (2018). Poor targeting: a gridded spatial analysis of the degree to which aid reaches the poor in Africa. World Dev..

[54] Deshpande AS, Mulat AK, Mao W, Diab MM, Ogbuoji O (2022). Coverage of social assistance in Ethiopia during the COVID-19 pandemic: A time-to-event analysis. BMJ Glob. Health.

[55] Tang CS (2007). Trajectory of traumatic stress symptoms in the aftermath of extreme natural disaster. J. Nerv. Ment. Dis..

[56] Díaz I, Williams N, Hoffman KL, Schenck EJ (2021). Nonparametric causal effects based on longitudinal modified treatment policies. J. Am. Stat. Assoc..

[57] van der Laan MJ, Rose S (2011). Targeted learning.

[58] Rudolph KE, Williams NT, Miles CH, Antonelli J, Diaz I (2023). All models are wrong, but which are useful? Comparing parametric and nonparametric estimation of causal effects in finite samples. J. Causal Inference.

[59] van der Laan MJ, Polley EC, Hubbard AE (2007). Super learner. Stat. Appl. Genet. Mol. Biol..

[60] van der Laan MJ, Gruber S (2012). Targeted minimum loss based estimation of causal effects of multiple time point interventions. Int. J. Biostat..

[61] López-Torres S, Pérez-Pedrogo C, Sánchez-Cardona I, Sánchez-Cesáreo M (2022). Psychometric properties of the PHQ-A among a sample of children and adolescents in Puerto Rico. Curr. Psychol..

[62] Nyongesa MK (2020). The reliability, validity and factorial structure of the Swahili version of the 7-item generalized anxiety disorder scale (GAD-7) among adults living with HIV from Kilifi Kenya. Ann. Gen. Psychiatry.

[63] de Man J (2021). Are the PHQ-9 and GAD-7 suitable for use in India? A psychometric analysis. Front. Psychol..

[64] Chibanda D (2016). Validation of screening tools for depression and anxiety disorders in a primary care population with high HIV prevalence in Zimbabwe. J. Affect. Disord..

[65] Arias de la Torre J (2021). Prevalence and variability of current depressive disorder in 27 European countries: a population-based study. Lancet Public Health.

[66] Tiirikainen K, Haravuori H, Ranta K, Kaltiala-Heino R, Marttunen M (2019). Psychometric properties of the 7-item generalized anxiety disorder scale (GAD-7) in a large representative sample of Finnish adolescents. Psychiatry Res..

[67] GBD 2019 Mental Disorders Collaborators (2022). Global, regional, and national burden of 12 mental disorders in 204 countries and territories, 1990–2019: a systematic analysis for the Global Burden of Disease Study 2019. Lancet Psychiatry.

[68] Shultz JM, Espinel Z, Galea S, Reissman DB, Ursano R, Fullerton C, Weisaeth L, Raphael B (2007). Disaster ecology implications for disaster psychiatry. Textbook of disaster psychiatry.

[69] Harville EW, Jacobs M, Boynton-Jarrett R (2015). When is exposure to a natural disaster traumatic? Comparison of a trauma questionnaire and disaster exposure inventory. PLoS One.

[70] Harville EW (2011). Combined effects of Hurricane Katrina and Hurricane Gustav on the mental health of mothers of small children. J. Psychiatr. Ment. Health Nurs..

[71] Ventevogel P, Jordans M, Reis R, de Jong J (2013). Madness or sadness? Local concepts of mental illness in four conflict-affected African communities. Confl. Health.

[72] Youngmann R, Minuchin-Itzigsohn S, Barasch M (1999). Manifestations of emotional distress among Ethiopian immigrants in Israel: patient and clinician perspectives. Transcult. Psychiatry.

[73] Roberts T (2020). “Is there a medicine for these tensions?” Barriers to treatment-seeking for depressive symptoms in rural India: A qualitative study. Soc. Sci. Med..

[74] Pedersen D, Tremblay J, Errázuriz C, Gamarra J (2008). The sequelae of political violence: assessing trauma, suffering and dislocation in the Peruvian highlands. Soc. Sci. Med..

[75] Hinton D, Hinton S, Thang P, Chau HA, Tran M (2003). “Hit by the wind” and temperature-shift panic among Vietnamese refugees. Transcult. Psychiatry.

[76] von Elm E (2007). The strengthening the reporting of observational studies in epidemiology (STROBE) statement: Guidelines for reporting observational studies. PLoS Med..

[77] Young Lives methods guide: the longitudinal survey. *University of Oxford* http://doc.ukdataservice.ac.uk/doc/5307/mrdoc/pdf/5307methods_guide_the_longitudinal_survey.pdf (2011).

[78] A guide to Young Lives research. *University of Oxford* www.younglives.org.uk (2017).

[79] Barnett I (2013). Cohort profile: The young lives study. Int. J. Epidemiol..

[80] Favara M (2021). Cohort profile update: The young lives study. Int. J. Epidemiol..

[81] Kroenke K (2009). The PHQ-8 as a measure of current depression in the general population. J. Affect. Disord..

[82] Spitzer RL, Kroenke K, Williams JBW, Löwe B (2006). A brief measure for assessing generalized anxiety disorder: the GAD-7. Arch. Intern. Med..

[83] Manzar MD (2021). Psychometric properties of the general anxiety disorders-7 scale using categorical data methods: a study in a sample of university attending Ethiopian young adults. Neuropsychiatr. Dis. Treat..

[84] Carroll HA (2020). Establishing reliability and validity for mental health screening instruments in resource-constrained settings: systematic review of the PHQ-9 and key recommendations. Psychiatry Res..

[85] Pham TN (2021). Utilization of mental health services among university students in Vietnam. Int. J. Ment. Health.

[86] Mughal AY (2020). A systematic review of validated screening tools for anxiety disorders and PTSD in low to middle income countries. BMC Psychiatry.

[87] Hengartner MP (2020). Is there a genuine placebo effect in acute depression treatments? A reassessment of regression to the mean and spontaneous remission. BMJ Evid. Based Med..

[88] Cerna-Turoff I, Chillrud LG, Rudolph KE, Casey JA (2023). Standards in responsibly sharing cohort data for transparency and reproducibility: Response to the Young Lives Study. Int. J. Epidemiol..

[89] Neumayer E, Plümper T (2007). The gendered nature of natural disasters: the impact of catastrophic events on the gender gap in life expectancy, 1981–2002. Ann. Assoc. Am. Geogr..

[90] Goldmann E, Galea S (2014). Mental health consequences of disasters. Annu. Rev. Public Health.

[91] Madley-Dowd P, Hughes R, Tilling K, Heron J (2019). The proportion of missing data should not be used to guide decisions on multiple imputation. J. Clin. Epidemiol..

[92] R Core Team. R: a language and environment for statistical computing. *R Foundation for Statistical Computing* https://www.R-project.org/ (2021).

[93] Williams N, Díaz I (2023). lmtp: an R package for estimating the causal effects of modified treatment policies. Obs. Stud..

[94] Rudolph KE (2022). When effects cannot be estimated: redefining estimands to understand the effects of naloxone access laws. J. Epidemiol..

