# Supplementary material for: Longitudinal patterns of natural hazard exposures and anxiety and depression symptoms among young adults in four low- and middle-income countries
Source: Sci Rep. 2024 May 8;14:10538. doi: 10.1038/s41598-024-60106-6 (PMC11078992; doi:10.1038/s41598-024-60106-6)
Supplement: Supplementary file 1 — Supplementary Information. [file 41598_2024_60106_MOESM1_ESM.docx]

Supplementary materials

[Supplementary Table S1. Frequencies and means of adjusted covariates in original and imputed data 2](#_Toc147850838)

[Supplementary Figure S1. Distribution of GAD-7 and PHQ-8 scores by severity 14](#_Toc147850839)

[Supplementary Table S2. STROBE checklist 18](#_Toc147850840)

[Supplementary Table S3. Natural hazard exposure by country and time point 36](#_Toc147850841)

[Supplementary Table S4. Targeted causal parameters for natural hazard exposure regimes 37](#_Toc147850842)

[Supplementary Table S5. List of covariates 38](#_Toc147850843)

**Title:** Longitudinal patterns of natural hazard exposures and anxiety and depression symptoms among young adults in four low- and middle-income countries.

**Journal:** Scientific Reports

**Authors:** Ilan Cerna-Turoff, Joan A. Casey, Katherine Keyes, Kara E. Rudolph, Daniel Malinsky

**Corresponding Author Email and Affiliation:** Dr. Ilan Cerna-Turoff ([it2208@caa.columbia.edu](mailto:it2208@caa.columbia.edu), Columbia University, Mailman School of Public Health, Department of Environmental Health Sciences, New York City, New York, USA)

# Supplementary Table S1. Frequencies and means of adjusted covariates in original and imputed data

Ethiopia

|  | | | |
| --- | --- | --- | --- |
|  |  | |  |
|  | Original *N* = 2299. Imputed *N* = 1766 | |  |
|  |  | Original dataset | Imputed dataset |
| **Individual-level covariates** |  |  |  |
| Young adult’s biological sex *n (%)* |  |  |  |
|  | Male | 1221 (53.1%) | 918 (52.0%) |
|  | Female | 1078 (46.9%) | 848 (48.0%) |
| Young adult’s ethnic group - Amhara *n (%)* |  |  |  |
|  | No | 1633 (71.0%) | 1261 (71.4%) |
|  | Yes | 665 (28.9%) | 505 (28.6%) |
| Young adult’s religion - Christian Orthodox *n (%)* |  |  |  |
|  | No | 651 (28.3%) | 587 (33.2%) |
|  | Yes | 1648 (71.7%) | 1179 (66.8%) |
| Young adult’s religion -  Islam *n (%)* |  |  |  |
|  | No | 1923 (83.6%) | 1444 (81.8%) |
|  | Yes | 376 (16.4%) | 322 (18.2%) |
| Young adult ever drank alcohol *n (%)* |  |  |  |
|  | No | 1957 (88.4%) | 1594 (90.3%) |
|  | Yes | 257 (11.6%) | 172 (9.7%) |
| Young adult’s recent subjective wellbeing score mean (SD) |  | 5.67 (1.61) | 5.62 (1.60) |
| Young adult almost died in infancy *n (%)* |  |  |  |
|  | No | 1671 (72.8%) | 1273 (72.1%) |
|  | Yes | 623 (27.2%) | 493 (27.9%) |
| Primary caregiver recently lost employment *n (%)* |  |  |  |
|  | No | 2146 (95.4%) | 1666 (94.3%) |
|  | Yes | 103 (4.6%) | 100 (5.7%) |
| Primary caregiver’s age mean (SD) |  | 30.29 (8.52) | 30.1(8.49) |
| Primary caregiver’s highest level of education *n (%)* |  |  |  |
|  | None | 1437 (62.5%) | 1039 (58.8%) |
|  | Some education | 851 (37.0%) | 727 (41.2%) |
| Primary caregiver’s relationship to the young adult *n (%)* - Biological  parent |  |  |  |
|  | No | 86 (3.7%) | 71 (4.0%) |
|  | Yes | 2211 (96.2%) | 1695 (96.0%) |
| Parents divorced in childhood *n (%)* |  |  |  |
|  | No | 1975 (92.3%) | 1630 (92.3%) |
|  | Yes | 165 (7.7%) | 136 (7.7%) |
| Father ill in childhood *n (%)* |  |  |  |
|  | No | 1483 (66.2%) | 1178 (66.7%) |
|  | Yes | 759 (33.9%) | 588 (33.3%) |
| Mother ill in childhood *n (%)* |  |  |  |
|  | No | 1279 (56.8%) | 997 (56.5%) |
|  | Yes | 972 (43.2%) | 769 (43.5%) |
| Mother died in childhood *n (%)* |  |  |  |
|  | No | 2204 (95.9%) | 1694 (95.9%) |
|  | Yes | 95 (4.1%) | 72 (4.1%) |
| Size of household mean (SD) |  | 5.43 (2.09) | 5.34 (2.05) |
| Food insecurity of household *n (%)* |  |  |  |
|  | We always eat enough of what we want | 150 (9.7%) | 164 (9.3%) |
|  | We eat enough but not always what we would like | 963 (62.0%) | 1103 (62.5%) |
|  | We sometimes do not eat enough | 396 (25.5%) | 427 (24.2%) |
|  | We frequently do not eat enough | 44 (2.8%) | 72 (4.1%) |
| Own land *n (%)* |  |  |  |
|  | No | 289 (18.7%) | 441 (25.0%) |
|  | Yes | 1253 (81.3%) | 1325 (75.0%) |
| **Community-level covariates before first time point** |  |  |  |
| Ever previous epidemic *n (%)* |  |  |  |
|  | No | 830 (51.3%) | 726 (41.1%) |
|  | Yes | 789 (48.7%) | 1040 (58.9%) |
| Population at t1 mean (SD) |  | 8005.98 (7501.46) | 9020 (7980.00) |
| Social worker in community at t1 *n (%)* |  |  |  |
|  | No | 1324 (57.6%) | 1030 (58.3%) |
|  | Yes | 975 (42.4%) | 736 (41.7%) |
| Theft/robbery at t1 *n (%)* |  |  |  |
|  | No | 947 (41.2%) | 688 (39.0%) |
|  | Yes | 1352 (58.8%) | 1078 (61.0%) |
| **Community-level covariates before second time point** |  |  |  |
| Population at t2 mean (SD) |  | 15873.86 (11646.66) | 18400 (11800.00) |
| Social worker in community at t2 *n (%)* |  |  |  |
|  | No | 939 (58.0%) | 907 (51.4%) |
|  | Yes | 680 (42.0%) | 859 (48.6%) |
| Theft/robbery at t2 *n (%)* |  |  |  |
|  | No | 995 (61.5%) | 960 (54.4%) |
|  | Yes | 624 (38.5%) | 806 (45.6%) |

India

|  | | | | |
| --- | --- | --- | --- | --- |
|  |  | |  | |
|  | Original *N* = 2704. Imputed *N* = 844 | |  | |
|  |  | Original dataset | | Imputed dataset |
| **Individual-level covariates** |  |  | |  |
| Young adult’s biological sex *n (%)* |  |  | |  |
|  | Male | 1403 (51.9%) | | 439 (52.0%) |
|  | Female | 1301 (48.1%) | | 405 (48.0%) |
| Young adult’s highest level of education *n (%)* -  Primary |  |  | |  |
|  | Primary | 806 (29.8%) | | 238 (28.2%) |
|  | Other education | 1387 (51.3%) | | 606 (71.8%) |
| Young adult’s highest level of education *n (%)* -  Secondary |  |  | |  |
|  | Secondary | 982 (36.3%) | | 406 (48.1%) |
|  | Other education | 1211 (44.8%) | | 438 (51.9%) |
| Young adult’s highest level of education *n (%)* -  University |  |  | |  |
|  | University | 296 (13.5%) | | 153 (18.1%) |
|  | Other education | 1897 (70.2%) | | 691 (81.9%) |
| Young adult’s ethnic group *n (%)* -  No caste |  |  | |  |
|  | No caste | 540 (19.97%) | | 211 (25.0%) |
|  | Caste | 2164 (80.0%) | | 633 (75.0%) |
| Young adult’s ethnic group *n (%)* -  Backwards  caste |  |  | |  |
|  | Backwards caste | 1250 (46.2%) | | 458 (54.3%) |
|  | Other | 1454 (53.8%) | | 386 (45.7%) |
| Young adult’s ethnic group *n (%) -*  Scheduled  caste |  |  | |  |
|  | Scheduled caste | 536 (19.8%) | | 134 (15.9%) |
|  | Other | 2168 (80.2%) | | 710 (84.1%) |
| Young adult’s primary language *n (%)* -  Telugu |  |  | |  |
|  | Telugu | 2237 (82.7%) | | 761 (90.2%) |
|  | Other | 462 (17.1%) | | 83 (9.8%) |
| Young adult’s religion *n (%)* |  |  | |  |
|  | Hindu | 2389 (88.4%) | | 771 (91.4%) |
|  | Other | 314 (11.6%) | | 73 (8.6%) |
| Young adult’s recent subjective wellbeing score mean (SD) |  | 5.04 (1.41) | | 5.14 (1.34) |
| Young adult almost died in infancy *n (%)* |  |  | |  |
|  | No | 2246 (83.1%) | | 683 (80.9%) |
|  | Yes | 458 (16.9%) | | 161 (19.1%) |
| Primary caregiver’s age mean (SD) |  | 26.14 (6.26) | | 25.90 (5.58) |
| Primary caregiver’s highest level of education *n (%)* |  |  | |  |
|  | None | 1802 (66.6%) | | 502 (59.5%) |
|  | Some | 901 (33.3%) | | 342 (40.5%) |
| Father ill in childhood *n (%)* |  |  | |  |
|  | No | 1939 (72.0%) | | 622 (73.7%) |
|  | Yes | 754 (28.0%) | | 222 (27.0%) |
| Mother ill in childhood *n (%)* |  |  | |  |
|  | No | 1895 (70.4%) | | 616 (73.0%) |
|  | Yes | 798 (29.6%) | | 228 (27.0%) |
| Size of household mean (SD) |  | 4.77 (1.81) | | 4.57 (1.67) |
| Own home *n (%)* |  |  | |  |
|  | No | 442 (16.4%) | | 162 (19.2%) |
|  | Yes | 2259 (83.6%) | | 682 (80.8%) |
| Own animals/livestock *n (%)* |  |  | |  |
|  | No | 1603 (59.33%) | | 540 (64.0%) |
|  | Yes | 1099 (40.67%) | | 304 (36.0%) |
| **Community-level covariates before first time point** |  |  | |  |
| Urban/rural *n (%)* |  |  | |  |
|  | No | 1857 (73.2%) | | 578 (68.5%) |
|  | Yes | 681 (26.8%) | | 266 (31.5%) |
| Population at t1 mean (SD) |  | 181.87 (101.74) | | 2270 (1500.00) |
| Social worker in community at t1 *n (%)* |  |  | |  |
|  | No | 2088 (83.0%) | | 647 (76.7%) |
|  | Yes | 427 (17.0%) | | 197 (23.3%) |
| **Community-level covariates before second time point** |  |  | |  |
| Population at t2 mean (SD) |  | 177.88 (94.79) | | 2270 (2470.00) |
| Social worker in community at t2 *n (%)* |  |  | |  |
|  | No | 992 (43.49%) | | 463 (54.9%) |
|  | Yes | 1289 (56.51%) | | 381 (45.1%) |

Peru

|  | | | |
| --- | --- | --- | --- |
|  |  | |  |
|  | Original *N* = 1921. *N* = 1232 | |  |
|  |  | Original dataset | Imputed dataset |
| **Individual-level covariates** |  |  |  |
| Young adult’s biological sex *n (%)* |  |  |  |
|  | Male | 968 (50.4%) | 601 (48.8%) |
|  | Female | 953 (49.6%) | 631 (51.2%) |
| Young adult’s religion *n (%)* -  None |  |  |  |
|  | Yes | 83 (4.3%) | 51 (4.1%) |
|  | No | 1837 (95.6%) | 1181 (95.9%) |
| Young adult ever smoked *n (%)* |  |  |  |
|  | No | 1503 (80.6%) | 994 (80.7%) |
|  | Yes | 361 (19.4%) | 238 (19.3%) |
| Young adult’s recent subjective wellbeing score mean (SD) |  | 6.34 (1.45) | 6.36 (1.46) |
| Young adult almost died in infancy *n (%)* |  |  |  |
|  | No | 1352 (70.4%) | 866 (70.3%) |
|  | Yes | 569 (29.6%) | 366 (29.7%) |
| Primary caregiver recently lost employment *n (%)* |  |  |  |
|  | No | 1727 (90.0%) | 1123 (91.2%) |
|  | Yes | 191 (10.0%) | 109 (8.8%) |
| Primary caregiver’s age mean (SD) |  | 28.81 (7.81) | 28.90 (8.23) |
| Primary caregiver’s highest level of education *n (%)* |  |  |  |
|  | None | 207 (10.8%) | 156 (12.7%) |
|  | Some | 1401 (72.9%) | 1076 (87.3%) |
| Parents divorced in childhood *n (%)* |  |  |  |
|  | No | 1621 (88.4%) | 1099 (89.2%) |
|  | Yes | 212 (11.6%) | 133 (10.8%) |
| Father ill in childhood *n (%)* |  |  |  |
|  | No | 1604 (84.7%) | 1049 (85.1%) |
|  | Yes | 290 (15.3%) | 183 (14.9%) |
| Mother ill in childhood *n (%)* |  |  |  |
|  | No | 1414 (74.5%) | 932 (75.6%) |
|  | Yes | 485 (25.5%) | 300 (24.4%) |
| Size of household mean (SD) |  | 5.04 (1.95) | 5.01 (1.95) |
| Food insecurity of household *n (%)* |  |  |  |
|  | We always eat enough of what we want | 599 (40.7%) | 501 (40.7%) |
|  | We eat enough but not always what we would like | 733 (49.8%) | 597 (48.5%) |
|  | We sometimes do not eat enough | 126 (8.6%) | 114 (9.3%) |
|  | We frequently do not eat enough | 15 (1.0%) | 20 (1.6%) |
| Own home *n (%)* |  |  |  |
|  | No | 447 (23.4%) | 256 (20.8%) |
|  | Yes | 1460 (76.6%) | 976 (79.2%) |
| Own land *n (%)* |  |  |  |
|  | No | 338 (23.0%) | 285 (23.1%) |
|  | Yes | 1129 (77.0%) | 947 (76.9%) |
| Own animals/livestock *n (%)* |  |  |  |
|  | No | 691 (46.4%) | 566 (45.9%) |
|  | Yes | 797 (53.6%) | 666 (54.1%) |
| **Community-level covariates before first time point** |  |  |  |
| Population at t1 mean (SD) |  | 145.85 (77.60) | 5080 (4220.00) |
| Youth crime at t1 *n (%)* |  |  |  |
|  | No | 828 (43.3%) | 589 (47.8%) |
|  | Yes | 1085 (56.7%) | 643 (52.2%) |
| Violent crime at t1 *n (%)* |  |  |  |
|  | No | 1134 (59.3%) | 785 (63.7%) |
|  | Yes | 779 (40.7%) | 447 (36.3%) |
| Theft/robbery at t1 *n (%)* |  |  |  |
|  | No | 286 (15.0%) | 195 (15.8%) |
|  | Yes | 1627 (85.0%) | 1037 (84.2%) |
| **Community-level covariates before second time point** |  |  |  |
| Population at t2 mean (SD) |  | 122 (69.01) | 14300 (34200.00) |
| Youth crime at t2 *n (%)* |  |  |  |
|  | No | 925 (57.8%) | 899 (73.0%) |
|  | Yes | 675 (42.2%) | 333 (27.0%) |
| Violent crime at t2 *n (%)* |  |  |  |
|  | No | 1119 (70.0%) | 1017 (82.5%) |
|  | Yes | 480 (30.0%) | 215 (17.5%) |
| Theft/robbery at t2 *n (%)* |  |  |  |
|  | No | 269 (16.9%) | 306 (24.8%) |
|  | Yes | 1327 (83.1%) | 926 (75.2%) |

Vietnam

|  | | | |
| --- | --- | --- | --- |
|  |  | |  |
|  | Original *N* = 2517. *N* = 1743 | |  |
|  |  | Original dataset | Imputed dataset |
| **Individual-level covariates** |  |  |  |
| Young adult’s biological sex *n (%)* |  |  |  |
|  | Male | 1243 (49.4%) | 870 (49.9%) |
|  | Female | 1274 (50.6%) | 873 (50.1%) |
| Young adult’s highest level of education *n (%)* |  |  |  |
|  | None | 898 (35.7%) | 658 (37.8%) |
|  | Primary or secondary | 1619 (64.3%) | 1085 (62.2%) |
| Young adult’s religion *n (%)* -  Minority religions |  |  |  |
|  | None | 2145 (85.25%) | 1897 (83.94%) |
|  | Ancestor worship | 92 (3.66%) | 89 (3.94%) |
|  | Buddhist | 194 (7.71%) | 191 (8.45%) |
|  | Cao Dai | 26 (1.03%) | 26 (1.15%) |
|  | Christian | 33 (1.31%) | 33 (1.46%) |
|  | Protestant | 25 (0.99%) | 23 (1.02%) |
|  | Other | 1 (0.04%) | 1 (0.04%) |
| Young adult’s religion *n (%)* -  Buddhist |  |  |  |
|  | Buddhist | 194 (7.7%) | 186 (10.7%) |
|  | Other | 2322 (92.3%) | 1557 (89.3%) |
| Young adult’s religion *n (%)* -  Minority religions |  |  |  |
|  | Minority religions | 85 (3.4%) | 1897 (5.7%) |
|  | Other | 2339 (92.9%) | 1644 (94.3%) |
| Young adult ever smoked *n (%)* |  |  |  |
|  | No | 1704 (90.2%) | 1541 (88.4%) |
|  | Yes | 184 (9.8%) | 202 (11.6%) |
| Young adult’s recent subjective wellbeing score mean (SD) |  | 5.86 (1.48) | 5.84 (1.52) |
| Young adult almost died in infancy *n (%)* |  |  |  |
|  | No | 2208 (87.7%) | 1505 (86.3%) |
|  | Yes | 309 (12.3%) | 238 (13.7%) |
| Primary caregiver’s age mean (SD) |  | 29.90 (7.48) | 29.8 (7.10) |
| Primary caregiver’s highest level of education *n (%)* |  |  |  |
|  | None | 701 (28.2%) | 614 (35.2%) |
|  | Some | 1789 (71.1%) | 1129 (64.8%) |
| Parents divorced in childhood *n (%)* |  |  |  |
|  | No | 2300 (95.2%) | 1659 (95.2%) |
|  | Yes | 115 (4.8%) | 84 (4.8%) |
| Father ill in childhood *n (%)* |  |  |  |
|  | No | 1819 (74.6%) | 1306 (74.9%) |
|  | Yes | 621 (25.4%) | 437 (25.1%) |
| Mother ill in childhood *n (%)* |  |  |  |
|  | No | 1832 (75.0%) | 1300 (74.6%) |
|  | Yes | 610 (25.0%) | 443 (25.4%) |
| Size of household mean (SD) |  | 4.13 (1.56) | 4.12 (1.59) |
| Food insecurity of household *n (%)* |  |  |  |
|  | We always eat enough of what we want | 816 (48.3%) | 730 (38.8%) |
|  | We eat enough but not always what we would like | 681 (40.3%) | 676 (38.8%) |
|  | We sometimes do not eat enough | 151 (8.9%) | 203 (11.6%) |
|  | We frequently do not eat enough | 41 (2.4%) | 134 (7.7%) |
| Own land *n (%)* |  |  |  |
|  | No | 75 (4.7%) | 298 (17.1%) |
|  | Yes | 1530 (95.3%) | 1445 (82.9%) |
| **Community-level covariates before first time point** |  |  |  |
| Ever previous epidemic *n (%)* |  |  |  |
|  | No | 1579 (78.3%) | 1319 (75.7%) |
|  | Yes | 439 (21.7%) | 424 (24.3%) |
| Population at t1 mean (SD) |  | 10217.48 (5705.91) | 9190 (4900.00) |
| Prostitution at t1 *n (%)* |  |  |  |
|  | No | 1930 (76.7%) | 1257 (72.1%) |
|  | Yes | 587 (23.3%) | 486 (27.9%) |
| Youth crime at t1 *n (%)* |  |  |  |
|  | No | 1685 (66.9%) | 1016 (58.3%) |
|  | Yes | 832 (33.1%) | 727 (41.7%) |
| Violent crime at t1 *n (%)* |  |  |  |
|  | No | 1752 (69.6%) | 1082 (62.1%) |
|  | Yes | 765 (30.4%) | 661 (37.9%) |
| **Community-level covariates before second time point** |  |  |  |
| Population at t2 mean (SD) |  | 10554.37 (4982.93) | 10200 (4990.00) |
| Prostitution at t2 *n (%)* |  |  |  |
|  | No | 1575 (78.1%) | 1218 (69.9%) |
|  | Yes | 443 (21.9%) | 525 (30.1%) |
| Youth crime at t2 *n (%)* |  |  |  |
|  | No | 1564 (77.5%) | 1222 (70.1%) |
|  | Yes | 454 (22.5%) | 521 (29.9%) |
| Violent crime at t2 *n (%)* |  |  |  |
|  | No | 837 (41.5%) | 542 (31.1%) |
|  | Yes | 1181 (58.5%) | 1201 (68.9%) |

*N* = total count of young adults; *n* = frequency; *SD* = standard deviation; t1 = time point 1; t2 = time point 2. Standard deviation rounded to two decimal places and frequencies to one decimal place. Slight discrepancies in total sample size and individual covariate counts in the original data are due to missingness. Descriptives for the imputed data are based upon a single complete data frame.

# Supplementary Figure S1. Distribution of GAD-7 and PHQ-8 scores by severity

Ethiopia


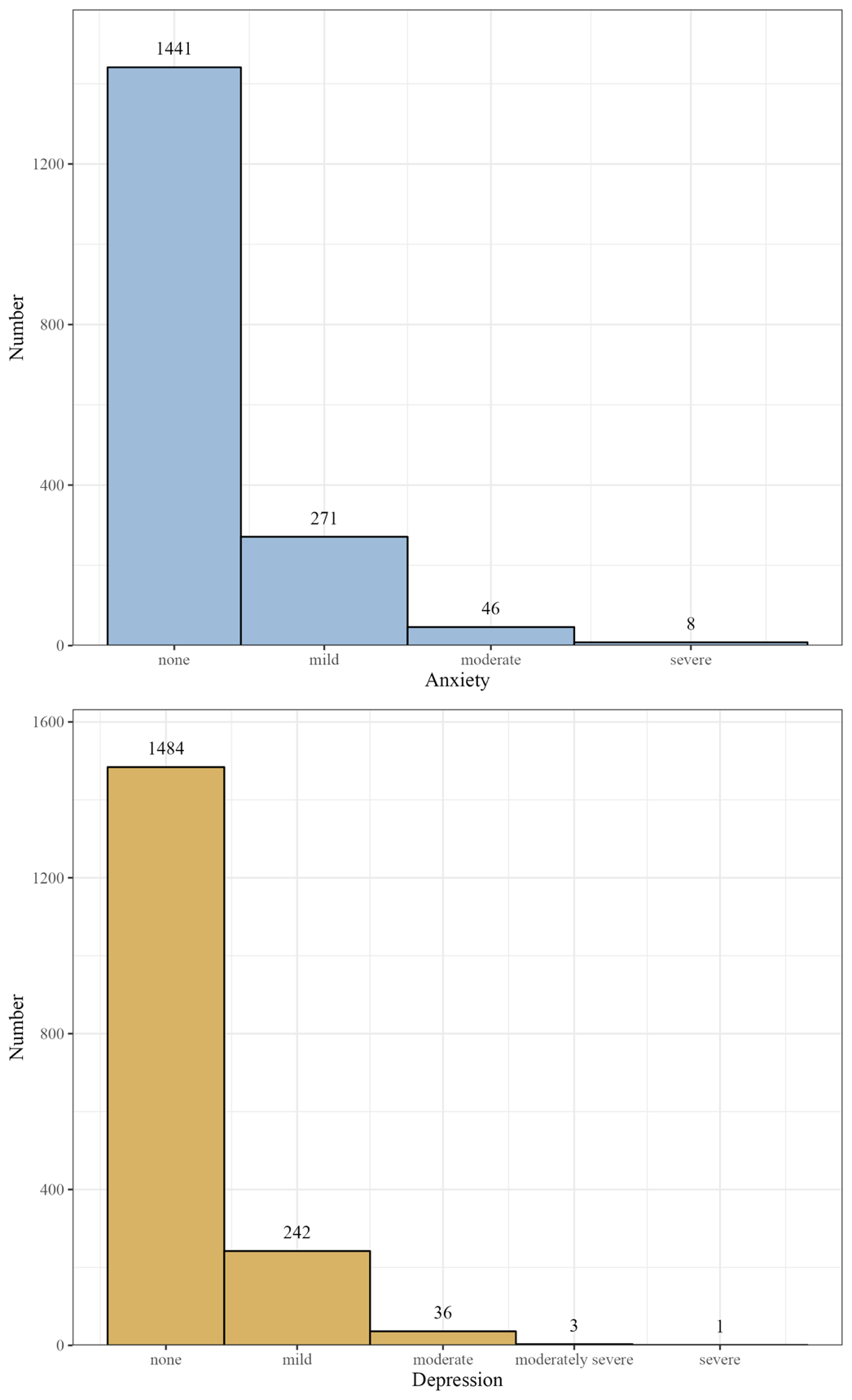


India


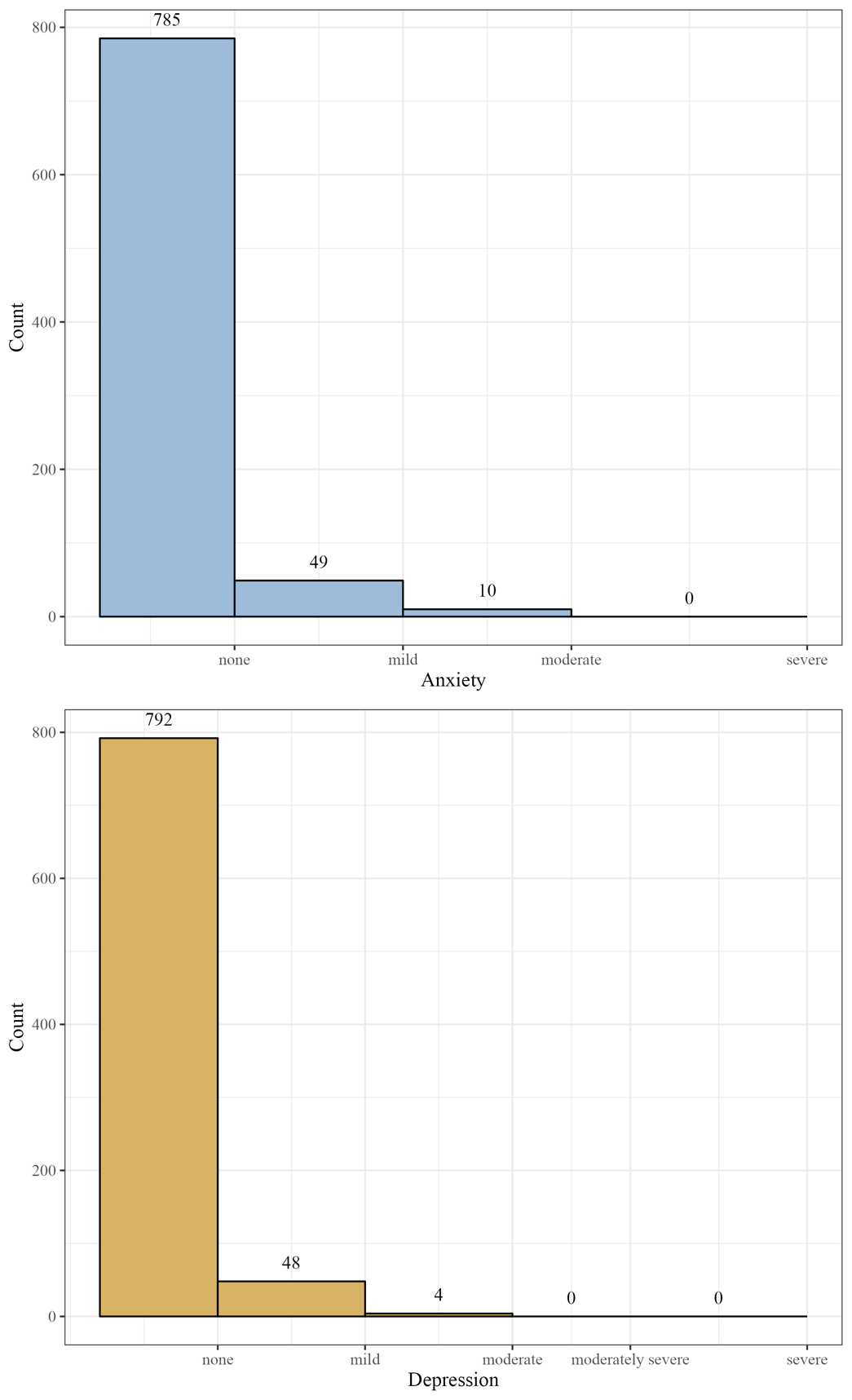


Peru


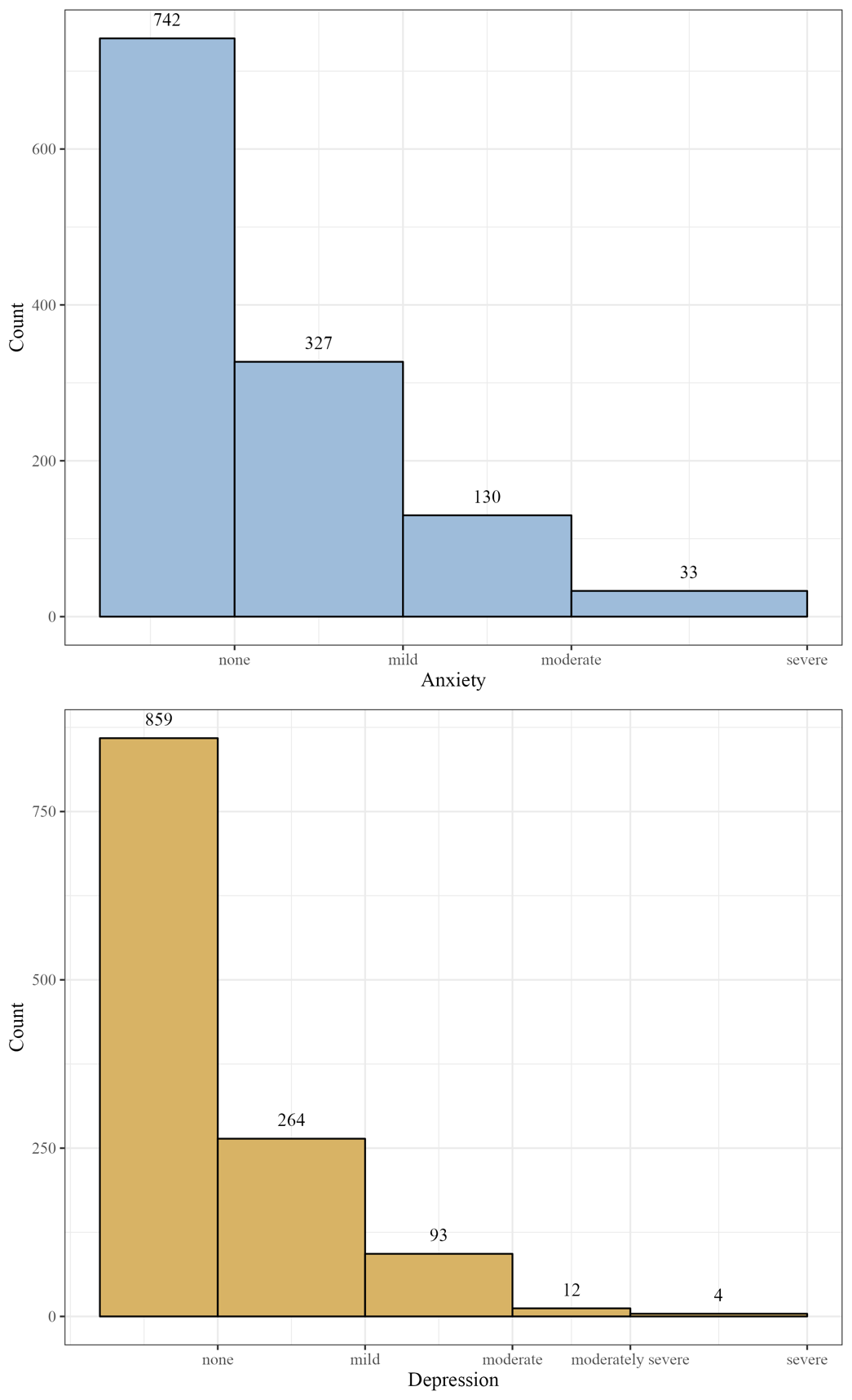


Vietnam


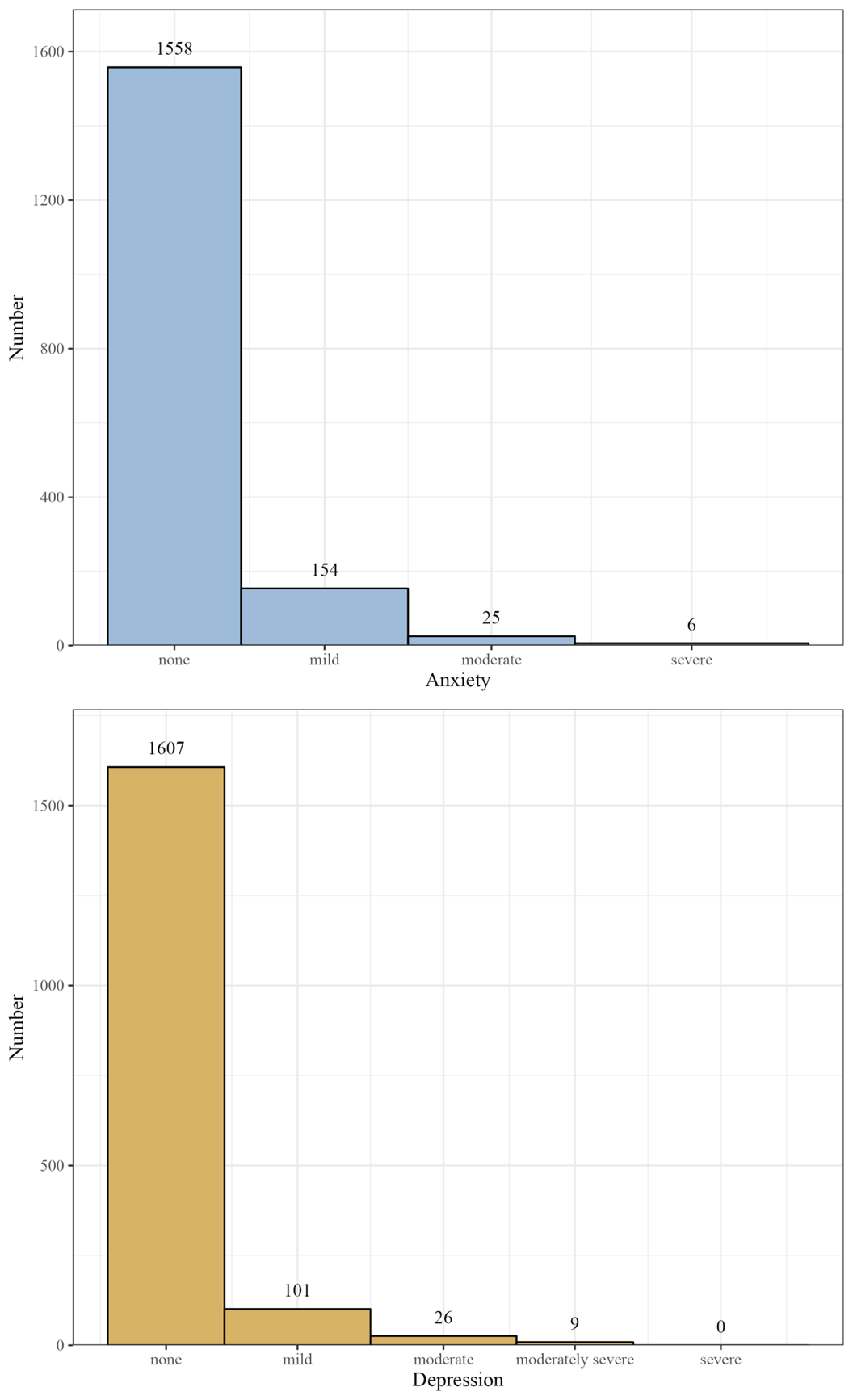


Severity thresholds are commonly applied when screening for anxiety and depression.

# Supplementary Table S2. STROBE checklist

|  | Item No | Recommendation | Page No. | Relevant text from manuscript |
| --- | --- | --- | --- | --- |
| **Title and abstract** | 1 | (*a*) Indicate the study’s design with a commonly used term in the title or the abstract | 1-2 | “Longitudinal patterns…”  “We applied a longitudinal targeted minimum loss-based estimator with an ensemble of machine learning algorithms for estimation.” |
|  |  | (*b*) Provide in the abstract an informative and balanced summary of what was done and what was found | 2 | **“**We analyzed longitudinal data (2002-2020) on 5585 young adults between 19 and 26 years in Ethiopia, India, Peru, and Vietnam. A binary question identified community-level exposure, and psychometrically validated scales measured recent anxiety and depression symptoms. Young adults with three exposure histories (“time point 1,” “time point 2,” and “both time points”) were contrasted with their unexposed peers. We applied a longitudinal targeted minimum loss-based estimator with an ensemble of machine learning algorithms for estimation. Young adults living in exposed communities did not exhibit substantially different anxiety or depression symptoms from their unexposed peers, except for young women in Ethiopia who exhibited less anxiety symptoms (average causal effect [ACE] estimate = -8.86 [95% CI: -17.04, -0.68] anxiety score). In this study, singular and repeated natural hazard exposures generally were not associated with later anxiety and depression symptoms.” |
| Introduction | | |  |  |
| Background/rationale | 2 | Explain the scientific background and rationale for the investigation being reported | 3-5 | “Natural hazards, such as tropical cyclones and earthquakes, are disruptive events that adversely affect populations globally. Over the past 20 years, natural hazards increased in their number and severity, claiming an estimated 1.23 million lives and affecting over four billion people [1]. A sizeable percentage of the affected population exhibits negative mental health symptoms and psychopathology after exposure to these events [2]. Associations between recent exposure to natural hazards and measures of impaired mental health have been found after the Mount Saint Helens volcanic eruption in the United States [3], the Canterbury earthquake in New Zealand [4], and a super-cyclone in India [5], among many other disasters. Younger individuals often exhibit worse mental health symptoms compared to adults [6]. Considering that natural hazards disproportionately affect low- and middle-income countries where the majority of the population is under the age of 30, a large segment of young people have the potential to experience negative mental health symptoms as a result of exposure [7,8].  Natural hazards may negatively affect mental health among young adults by either giving rise to molecular changes that increase susceptibility to mental health conditions or by changing the socioecological environment [9–12]. Life course epidemiology posits that specific developmental periods and transition points are sensitive and therefore, can exert a greater influence on later health [13]. In particular, early childhood and adolescence are two critical stages of development [14]. From birth until adolescence, the brain undergoes a rapid period of development, and high levels of neuroplasticity increase susceptibility to environmental stressors [15]. The body responds to stress by producing a glucocorticoid, cortisol, which can pass through the blood-brain barrier to shape neural development. As outlined by Tottenham and Sheridan [16], an extensive body of animal and human studies document how cortisol alters the size, complexity, and activity of receptors in the brain that control socioemotional functioning. Stress-related brain structures develop at different stages, plateauing after the age of 25 years, so environmental exposures at several points in childhood, adolescence, and young adulthood are likely to be important for mental health over the life course [16]. Studies of hurricanes [9] and earthquakes [10,11] have found that children and adults experience changes in brain function and structure after exposure, which relate to stress and emotional reactivity in young adulthood and later life.  Social epidemiology proposes that natural hazards alter the context where a child lives and grows. Changes to the socioecological environment may lead to ongoing disadvantage that engenders the development of poor mental health in young adulthood [12]. After natural hazards, children may experience long-term separation from family members, displacement to new communities, or familial strain resulting from economic losses of their home or livelihood. Hurricane Katrina, for instance, produced separation of household heads and adult children at 2.2 and 2.7 times the national rate in a one-year period after exposure [17]. Amongst low-income parents, those who were relocated to new communities and those who were unstably housed had more psychological distress and perceived stress than returned households [18]. New or exacerbated economic vulnerability places children and adolescents at increased risk of child labor [19], early marriage [20], and other negative circumstances that can worsen their mental health and general wellbeing. Individual and household level vulnerability is further influenced by the overall economic context. Rubens et al. [21] found that natural hazards were more strongly correlated with internalizing and externalizing among young people in countries with a medium Human Development Index (HDI) ($r$ = 0.56) versus high HDI ($r$ = 0.15) [21]. Regardless of context, gender has consistently been identified as an important factor, as girls tend to have worse mental health symptoms following natural hazard exposure than boys [6,21–23].”  Natural hazards are inherently multidimensional in that they have geophysical, atmospheric, hydrological, or other drivers that lead to multiple traumatic experiences during a disaster event. Spatial variability may also occur, even within small geographic areas. A lack of consensus exists in disaster studies regarding how to measure exposure. In practice, exposure has been operationalized as a series of questions of individual traumatic experiences to form scales, a continuous metric of area-level severity based upon aerial or sensor-based measures, a binary measure of exposure within administrative areas were the natural hazard occurred, or a binary measure of self-reported or community exposure [24]. Binary exposure measures on the community level are commonly used in low- and middle-income countries and serve as the exposure metric for this study [25–30]. Community-level exposures are then assigned to individuals. The socioecological model has been evoked as the theoretical basis for the validity of this exposure assignment, which posits that natural hazards change one’s environment and by way of this change, impact individuals [12,31]. |
| Objectives | 3 | State specific objectives, including any prespecified hypotheses | 5-6 | “This study aims to address these gaps by estimating how three patterns of natural hazard exposure histories influence later anxiety and depression symptoms among young adults in four low- and middle-income countries. Given the importance of gender in mental health symptoms, we further seek to estimate how exposure patterns may differ among young women and men.” |
| Methods | | |  |  |
| Study design | 4 | Present key elements of study design early in the paper | 2 | “We applied a longitudinal targeted minimum loss-based estimator with an ensemble of machine learning algorithms for estimation.” |
| Setting | 5 | Describe the setting, locations, and relevant dates, including periods of recruitment, exposure, follow-up, and data collection | 16 | “The Young Lives study followed a cohort of children from birth to young adulthood in Ethiopia, India, Peru, and Vietnam from 2002 to the present [78]. Five waves of data collection occurred at regular intervals every three to four years from 2002 to 2016.” |
| Participants | 6 | (*a*) Give the eligibility criteria, and the sources and methods of selection of participants. Describe methods of follow-up | 16, 17 | “Sentinel sites were selected from poorer regions of each country using a three-stage sampling frame [77].”  “The young adults were recruited as children as well as their adult caregivers. Within sentinel sites, households containing children within the target age group were randomly selected [77].”  “A purposeful sample of community respondents was selected at each wave by national field team supervisors. Community respondents included teachers, government officials, and other local leaders [77].” |
|  |  | (*b*) For matched studies, give matching criteria and number of exposed and unexposed | 6, 18 | “Our study design therefore had three possible exposure regimes: (a) “time point 1”: exposure to one or more natural hazards in 2002 when participants were under eight years of age; (b) “time point 2”: exposure to one or more natural hazards in 2016 when participants were 15 and 22 years old, respectively; and (c) “both time points”: exposure to one or more natural hazards in 2002 and 2016.”  “A natural hazard occurred in most young adult’s communities (68.3%) at the measured time points.” |
| Variables | 7 | Clearly define all outcomes, exposures, predictors, potential confounders, and effect modifiers. Give diagnostic criteria, if applicable | 17-19 | **“Definition of exposure**  Community respondents were asked a series of binary questions on exposure to different natural hazards at each survey wave between 2002 until 2016 (*“In the past [number of years since last survey round], has any of the following natural disasters occurred in this locality?”*). The original baseline survey in 2002 asked community respondents about natural disasters in the past two years. We organized the responses into ten non-overlapping categories of geophysical and climactic natural hazards and a category of no exposure (see **Supplementary Tab. S3**). Due to inconsistent documentation of natural hazard types at each survey wave and the potential for duplicate reporting of the same disaster event by different community respondents, we aggregated exposure across types to create a binary exposure (i.e., a community was exposed if one or more natural hazards were reported and unexposed if no natural hazard was reported). Each young adult was then assigned a community exposure based upon consistently living in a community since the last survey wave. We further restricted exposure to two survey waves that were meaningful stages in child development and collected consistent exposure information—the first survey wave in 2002 and the fifth survey wave in 2016. Our study design therefore had three possible exposure regimes: (a) “time point 1”: exposure to one or more natural hazards in 2002 when participants were under eight years of age; (b) “time point 2”: exposure to one or more natural hazards in 2016 when participants were 15 and 22 years old, respectively; and (c) “both time points”: exposure to one or more natural hazards in 2002 and 2016.  Expected mental health scores were estimated for participants under each of these potential regimes and contrasted to a regime in which individuals lived in communities that were never exposed to natural hazards. The causal contrasts of interest were the expected differences in mental health scores under each exposure regime versus never being exposed. These estimates may be interpreted as average causal effects (ACEs) under the assumption that there were no unmeasured sources of confounding (see **Supplementary Tab. S4**) [56].  **Definition of outcomes**  Young adults reported their recent anxiety and depression symptoms in two of the three rounds of telephone surveys in 2020, using psychometrically validated scales—the Generalized Anxiety Disorder-7 (GAD-7) and the Patient Health Questionnaire depression-8 (PHQ-8). The PHQ-8 is an eight-question screener that measures depressive incidents in the past two weeks [81], and the GAD-7 is a seven-item questionnaire that measures symptoms of anxiety in the past two weeks [82]. Both tools have a strong overlap with the Diagnostic and Statistical Manual of Mental Disorders (DSM-IV) criteria [81,82]. They have been extensively used globally with young adults and have been validated in the four study countries [83–86]. GAD-7 and PHQ-8 scores were collected twice; we sought to avoid possible biases from regression towards the mean often exhibited in repeat psychological testing and seasonal differences in mental health symptoms by analyzing the first time that mental health scores were recorded [87]. Only participants who answered all questions in the series were included in the analysis to maintain the psychometric validity of the mental health scales (percentage of participants missing items for GAD-7 and PHQ-8 scales: less than 1.0% for Ethiopia, India, and Vietnam and approximately 1.7% for Peru).  **Covariates**  Based on epidemiological theory about the correlates of mental health outcomes and factors related to natural hazard exposure, we included a wide selection of individual-, household-, and community-level covariates (see **Supplementary Tab. S5**). Additional information on data preparation can be found in previous work [88]. Gender was a key covariate included in the outcome models to reflect that women tend to have worse mental health symptoms after exposure to disaster events [89,90]. Covariates varied in their percentage of missingness from less than 1.0% to 36.3%. For all missing covariates, we conducted multiple imputation to analyze the dataset as if it was complete, in line with past recommendations [91].” |
| Data sources/ measurement | 8* | For each variable of interest, give sources of data and details of methods of assessment (measurement). Describe comparability of assessment methods if there is more than one group | 16, Table 4 | “Our study merged information from surveys administered to adult caregivers, community respondents, and young adults (**Table 4**).” |
| Bias | 9 | Describe any efforts to address potential sources of bias | 19, 20 | “GAD-7 and PHQ-8 scores were collected twice; we sought to avoid possible biases from regression towards the mean often exhibited in repeat psychological testing and seasonal differences in mental health symptoms by analyzing the first time that mental health scores were recorded [87]. Only participants who answered all questions in the series were included in the analysis to maintain the psychometric validity of the mental health scales…”  “To reduce the extent of practical positivity violations, we restricted analyses to geographic areas where the risk of natural hazards was more varied, conditional on covariates (Ethiopia: excluded desert communities [29.9%], India: excluded hill communities and regions that consisted only of inland plains [67.4%], and Vietnam: excluded the Red River region [22.9%]). In the excluded areas, the participants were always exposed to natural hazards (with no “unexposed” comparison peers), and therefore, these regions violated the positivity identification assumption. We also restricted the data to those individuals who did not move to different communities between the first and second exposure time points to reduce potential exposure misclassification. We incorporated community clustering for standard error calculation.” |
| Study size | 10 | Explain how the study size was arrived at | 19 | “Only participants who answered all questions in the series were included in the analysis to maintain the psychometric validity of the mental health scales (percentage of participants missing items for GAD-7 and PHQ-8 scales: less than 1.0% for Ethiopia, India, and Vietnam and approximately 1.7% for Peru).” |
| Quantitative variables | 11 | Explain how quantitative variables were handled in the analyses. If applicable, describe which groupings were chosen and why | 17-18, 19 | “Community respondents were asked a series of binary questions on exposure to different natural hazards at each survey wave between 2002 until 2016 (*“In the past [number of years since last survey round], has any of the following natural disasters occurred in this locality?”*). The original baseline survey in 2002 asked community respondents about natural disasters in the past two years. We organized the responses into ten non-overlapping categories of geophysical and climactic natural hazards and a category of no exposure (see **Supplementary Tab. S3**). Due to inconsistent documentation of natural hazard types at each survey wave and the potential for duplicate reporting of the same disaster event by different community respondents, we aggregated exposure across types to create a binary exposure (i.e., a community was exposed if one or more natural hazards were reported and unexposed if no natural hazard was reported). Each young adult was then assigned a community exposure based upon consistently living in a community since the last survey wave. We further restricted exposure to two survey waves that were meaningful stages in child development and collected consistent exposure information—the first survey wave in 2002 and the fifth survey wave in 2016. Our study design therefore had three possible exposure regimes: (a) “time point 1”: exposure to one or more natural hazards in 2002 when participants were under eight years of age; (b) “time point 2”: exposure to one or more natural hazards in 2016 when participants were 15 and 22 years old, respectively; and (c) “both time points”: exposure to one or more natural hazards in 2002 and 2016.”  “GAD-7 and PHQ-8 scores were collected twice; we sought to avoid possible biases from regression towards the mean often exhibited in repeat psychological testing and seasonal differences in mental health symptoms by analyzing the first time that mental health scores were recorded [87]. Only participants who answered all questions in the series were included in the analysis to maintain the psychometric validity of the mental health scales (percentage of participants missing items for GAD-7 and PHQ-8 scales: less than 1.0% for Ethiopia, India, and Vietnam and approximately 1.7% for Peru).”  “Based on epidemiological theory about the correlates of mental health outcomes and factors related to natural hazard exposure, we included a wide selection of individual-, household-, and community-level covariates (see **Supplementary Tab. S5**)” |
| Statistical methods | 12 | (*a*) Describe all statistical methods, including those used to control for confounding | 19-20 | **“Analyses**  We applied a longitudinal targeted minimum loss-based estimator to estimate anxiety and depression scores under exposure regimes, using the lmtp package in the R statistical software [92,93]. This estimator is doubly robust and non-parametric [56]. We fit the models for treatment and outcome using an ensemble of machine learning algorithms in SuperLearner [59]. The ensemble included the sample mean, generalized linear models, tree-based algorithms (extreme gradient boosting), and spline methods (multivariate adaptive regression splines and general additive models) and used five-fold cross-validation to select an optimal combination. We further added trimming at the 99^th^ percentile to exclude extreme weights in estimation. An analysis stratified by gender was conducted to identify differential effects.  The lmtp package allows for specification of covariates separately in exposure, censoring, and outcome models to mitigate practical positivity violations [94]. To reduce the extent of practical positivity violations, we restricted analyses to geographic areas where the risk of natural hazards was more varied, conditional on covariates (Ethiopia: excluded desert communities [29.9%], India: excluded hill communities and regions that consisted only of inland plains [67.4%], and Vietnam: excluded the Red River region [22.9%]). In the excluded areas, the participants were always exposed to natural hazards (with no “unexposed” comparison peers), and therefore, these regions violated the positivity identification assumption. We also restricted the data to those individuals who did not move to different communities between the first and second exposure time points to reduce potential exposure misclassification. We incorporated community clustering for standard error calculation.” |
|  |  | (*b*) Describe any methods used to examine subgroups and interactions | 20 | “An analysis stratified by gender was conducted to identify differential effects.” |
|  |  | (*c*) Explain how missing data were addressed | 19 | “For all missing covariates, we conducted multiple imputation to analyze the dataset as if it was complete, in line with past recommendations [91].” |
|  |  | (*d*) If applicable, explain how loss to follow-up was addressed | 17 | “Of the original sample of 11,784 children, 83.7% of the participants were retained across survey waves and into the 2020 telephone surveys [80].” |
|  |  | (*e*) Describe any sensitivity analyses | - | - |
| Results | | |  |  |
| Participants | 13* | (a) Report numbers of individuals at each stage of study—eg numbers potentially eligible, examined for eligibility, confirmed eligible, included in the study, completing follow-up, and analysed | 6, 19 | “Only participants who answered all questions in the series were included in the analysis to maintain the psychometric validity of the mental health scales (percentage of participants missing items for GAD-7 and PHQ-8 scales: less than 1.0% for Ethiopia, India, and Vietnam and approximately 1.7% for Peru).”  “A total of 5585 young adults were included in the final analysis (Ethiopia: 1766, India: 844, Peru: 1232, and Vietnam: 1743) who lived in 158 communities (Ethiopia: 19, India: 39, Peru: 75, and Vietnam: 25).” |
|  |  | (b) Give reasons for non-participation at each stage | - | - |
|  |  | (c) Consider use of a flow diagram | - | - |
| Descriptive data | 14* | (a) Give characteristics of study participants (eg demographic, clinical, social) and information on exposures and potential confounders | 6-7, Fig S1, Table 2, Fig 1 | “A total of 5585 young adults were included in the final analysis (Ethiopia: 1766, India: 844, Peru: 1232, and Vietnam: 1743) who lived in 158 communities (Ethiopia: 19, India: 39, Peru: 75, and Vietnam: 25). The distribution of covariates remained similar before and after imputation of the missing data (see **Supplementary Tab. S1**). After excluding communities for practical positivity violations, the most commonly reported natural hazard types were drought for Ethiopia (36.4%) and India (45.9%); unspecified types of natural hazards in Peru (28.9%); and flooding in Vietnam (30.0%) (**Fig. 1**). A natural hazard occurred in most young adult’s communities (68.3%) at the measured time points. Comparing across countries, the percentages ranged from 57.7% exposed in Peru to 88.2% exposed in Vietnam. Communities were likewise frequently exposed to more than one natural hazard, with Vietnam having the highest average of 2.5 natural hazards per community.  Most GAD-7 and PHQ-8 scores were low in the final sample used for analysis (**Table 1**), with exception of Peru which had average and median scores that were in the mild range for anxiety and depression symptoms (see **Supplementary Fig. S1**).” |
|  |  | (b) Indicate number of participants with missing data for each variable of interest | 19, Table S1 | “Only participants who answered all questions in the series were included in the analysis to maintain the psychometric validity of the mental health scales (percentage of participants missing items for GAD-7 and PHQ-8 scales: less than 1.0% for Ethiopia, India, and Vietnam and approximately 1.7% for Peru).” |
|  |  | (c) Summarise follow-up time (eg, average and total amount) | 16, 17 | “The Young Lives study followed a cohort of children from birth to young adulthood in Ethiopia, India, Peru, and Vietnam from 2002 to the present [78]. Five waves of data collection occurred at regular intervals every three to four years from 2002 to 2016.”  “The sixth wave of data collection was adapted for the COVID-19 pandemic. In 2020, a series of three rounds of telephone surveys were conducted, during which young adults self-reported on their recent anxiety and depression symptoms. The participants were young adults in 2020, aged 19 and 26 years. Of the original sample of 11,784 children, 83.7% of the participants were retained across survey waves and into the 2020 telephone surveys [80].” |
| Outcome data | 15* | Report numbers of outcome events or summary measures over time | 18 | “Young adults reported their recent anxiety and depression symptoms in two of the three rounds of telephone surveys in 2020, using psychometrically validated scales—the Generalized Anxiety Disorder-7 (GAD-7) and the Patient Health Questionnaire depression-8 (PHQ-8). The PHQ-8 is an eight-question screener that measures depressive incidents in the past two weeks [81], and the GAD-7 is a seven-item questionnaire that measures symptoms of anxiety in the past two weeks [82].” |
| Main results | 16 | (*a*) Give unadjusted estimates and, if applicable, confounder-adjusted estimates and their precision (eg, 95% confidence interval). Make clear which confounders were adjusted for and why they were included | 7-8, Table 2, Table S1 | “**Table 2** presents the average differences between young adults with each exposure pattern as compared to young adults who lived in communities that were never exposed. In the full sample, no pattern of exposure was associated with increases in anxiety and depression symptoms. Some estimates (e.g., repeat exposure in Vietnam) exhibited wide confidence intervals that illustrate a substantial uncertainty in the effect estimates of these exposure patterns in this dataset.” |
|  |  | (*b*) Report category boundaries when continuous variables were categorized | S6 | - |
|  |  | (*c*) If relevant, consider translating estimates of relative risk into absolute risk for a meaningful time period | - | - |
| Other analyses | 17 | Report other analyses done—eg analyses of subgroups and interactions, and sensitivity analyses | 8, Table 3 | “When examining young men and women separately, a more nuanced pattern of mental health emerges. Ethiopian young women who lived in communities that were repeatedly exposed to natural hazards had GAD-7 scores that were 8.86 points lower [95% CI: -17.04, -0.68] than young women who had never been exposed (*p*-value = 0.034). A difference in PHQ-8 and GAD-7 scores were not identified in any other country or gender subgroup (**Table 3**).” |
| Discussion | | |  |  |
| Key results | 18 | Summarise key results with reference to study objectives | 8-12 | **“Discussion**  Young adults who lived in communities that were exposed to any natural hazard exposure pattern did not have substantially different GAD-7 and PHQ-8 scores from their unexposed peers. Prior exposure was not associated with increased risk of later anxiety and depression symptoms in young adulthood in these four countries, regardless of context, and effects were not magnified when young people were exposed at both time points. Past studies have found mixed associations between exposure to natural hazards and mental health, likely due to differences in exposure measurement, study population, and study design. A global meta-analysis contrasting disaster exposed to unexposed groups and incorporating pre-post testing found slight increases in depression among the exposed population (standardized mean difference [SMD] = 0.55, 95% CI: 0.05-1.06). Anxiety symptoms, however, did not conclusively differ [38]. Specific to children, Norris et al. [6] found mixed but generally worse mental health symptoms among younger age groups as compared to adults across multiple studies. Another meta-analytic study similarly estimated a slight increase in combined anxiety and depression metrics after exposure for youth ($r$ = 0.18, 95% CI: 0.14, 0.22) [21], and a recent systematic review of post-disaster mental health recovery from 29 countries found that depression remained elevated for years after natural disaster or pandemic exposure among young people and at a higher rate than in adult populations [39]. Amongst these studies, exposure was commonly measured in low- and middle-income countries as residence in a geographic area affected by the natural hazard (e.g., [25–30]). The current literature tends to indicate that exposure to natural hazards induces a slight but lasting increase in depression and anxiety symptoms among young people, with additional variation depending on the severity, duration, and personal impact of the natural hazard. In contrast, this study generally did not find evidence that exposure to natural hazards related to anxiety or depression symptoms. The findings support possible conclusions about the frequency and historical/longitudinal patterns of exposure (i.e., repeat exposure, exposure in adolescence/young adulthood, and exposures in early life) which are not typical of studies that examine one natural hazard event, and they complement existing evidence involving “dose” of exposure from proximity or severity to natural hazards.  The type and severity of traumatic experiences over one’s lifetime and during the natural hazard may be most predictive in determining who experiences poor mental health. Tang et al. [40] found that across studies of children, depression symptoms only significantly increased among subgroups that had prior experiences of trauma or specific negative experiences during and after the natural hazard (i.e., being trapped during the disaster; experiencing injury, fear, or bereavement during the disaster; witnessing injury or death; or having poor social support). Personal behaviors and worldviews additionally act to protect or exacerbate symptoms. Stoicism and maladaptive coping, such as venting and distraction, can lead to greater deterioration of mental health, whereas acceptance, positive reframing, and humor are protective [41]. Similarly, individuals that use religious coping to find meaning and positive lessons in negative events often have overall better mental health [42]. Young people, likewise, are embedded within families, communities, and societies that influence their mental health symptoms and recovery. Familial and social support as well as the overall sociopolitical context influence the development of symptoms of poor mental health after natural hazards [21,43,44]. This nuance collectively points to a complicated pattern of individual and socioecological factors that may modify the relationship between natural hazards and mental health. Although our study sought to adjust for all meaningful pre-exposure covariates in the dataset, individual, relational, and societal dimensions of mental health response are not fully understood or sufficiently captured in data on disaster-related mental health. Furthermore, a lack of consensus exists on how to best operationalize exposure, and measures of exposure at the community or individual level may differ in their sensitivity. Exposure has been constructed using individual disaster-related stressors or area-level measures of proximity, severity, and general residence in an affected area [24]. These measurements merit further exploration among young people in future research.  Unexpectedly, young women in Ethiopia exhibited lower GAD-7 scores (less reported anxiety) when living in communities that were repeatedly exposed to natural hazards at both time points. The literature largely finds that women have worse mental health symptomology than men when exposed to natural hazards [6,21–23]. Ethiopia is likely no exception. Of the limited evidence, a cross-sectional study from the capital of Addis Ababa found that woman had 1.74 (95% CI: 1.21, 2.50) higher adjusted odds of posttraumatic stress disorder (PTSD) after a landslide [45]. School-based samples among adolescents and young adults from various parts of the country further indicate that female gender is associated with higher depression and anxiety symptoms [46–48]. The lower anxiety symptoms among repeatedly exposed young women in Ethiopia may be an artifact of unmeasured confounding from factors that were not included in this study. One such covariate would be the structure of disaster relief aid. It is plausible that a greater amount of gender-sensitive aid was allocated to communities where natural hazards repeatedly occurred, as compared to communities that did not experience natural hazards. Ethiopia receives some of the world’s highest amount of foreign aid and hosts several national safety net programs that provide gender-responsive support to households affected by drought and other climate-related shocks [49,50]. It may be that young women in affected communities benefited from this investment in early childhood disproportionate to their peers and therefore, had lower anxiety for their future wellbeing when repeated natural hazards occurred. When baseline services are not well-distributed nationally, and one group receives targeted aid, asymmetries commonly occur [51]. Ethiopia has had mixed success in its aid distribution and gender-responsiveness [52,53]; however, qualitative evidence collected as part of a longitudinal study with Ethiopian adolescents and young adults, aged 10 to 20 years, corroborates that climate mitigation strategies led to investment in gender-sensitive social protection programs, which positively impacted young women and girls [50]. Young women who lived in communities where these programs were implemented may have benefited over the long-term. Furthermore, the study measured anxiety and depression during the height of the COVID-19 pandemic. Poor households in rural areas were 88.0% more likely to receive government assistance within the 10 months after the start of the pandemic than non-poor households. These households were disproportionately female headed and overlapped with areas of the country where natural hazards frequently occur [54]. COVID-19 investment could have lowered recent anxiety symptoms among young women in these communities. One’s perception of the government’s response to natural hazards may be as important as the actual aid received in influencing mental health symptoms. Although a different context, an estimated 18.0% of survivors of the Southeast Asian earthquake and tsunami in Thailand newly developed posttraumatic stress symptoms when they perceived that there was a low level of government support [55].” |
| Limitations | 19 | Discuss limitations of the study, taking into account sources of potential bias or imprecision. Discuss both direction and magnitude of any potential bias | 13-15 | “Our study also has several limitations. Community respondents reported on community-level exposure to natural hazards, which does not fully capture individual experiences of young adults during disaster events or discordance between individuals and community members in their reporting. Certain types of natural hazards may affect a segment of the community, leading to differential exposure to the natural hazard within the same area (e.g., the difference between living on hilltops versus valleys when community flooding occurs). This analysis should therefore be interpreted as estimating differences in the average mental health of young adults living in communities that experienced and did not experience natural hazards. Substantial debate exists as to the best way to measure exposure to natural hazards, but it is widely acknowledged that living in communities affected by natural hazard will disrupt the socioecological environment to some extent, regardless of the severity of personal experiences, and community exposure is deemed a valid metric in the disaster literature [24,68]. Relatedly, information on individual experiences during natural hazards was not captured in the Young Lives study. Specific experiences—namely, damage to one’s house and belongings, perceived or experienced danger, and illness or injury to one’s self or others—in validated trauma scales are most indicative of poor mental after natural hazard exposure [69,70]. We did not have access to validated scales to contrast individual experiences, but we hypothesize that it would have been more sensitive than binary reporting of community exposures. Alternative metrics for measuring exposure on a continuous scale, such as strength of, proximity to, or damages from a natural hazard, would capture how specific levels or severity of exposure influenced mental health outcomes. It may be that mental health worsens as proximity and impact of exposure increases. Nevertheless, binary measures of total community exposure can estimate the overall effect in the target population [24]. Furthermore, recall bias may exist in exposure reporting. Although community respondents are likely to remember major natural hazards, they may have underreported minor natural hazards, which would artificially make the exposed and unexposed groups seem more similar than they were in actuality and bias towards the null. In addition, this dataset did not consistently collect information on exposure types at each wave, which precluded analysis of the influence of specific types of natural hazards or the creation of a count for the number of natural hazards that occurred in a community. It may also be that other developmental periods are more influential on mental health in young adulthood. The two time periods used in this study, however, are emphasized in global health strategies for intervening on lifelong health [14], and other measures were not comparable. Last, we were interested in distal exposures that happened in early childhood and/or four years prior to the outcome measurement. For the majority of individuals who exhibit poor mental health after natural hazard exposure, symptoms are short lived and improve over time [6,21,23]. It may be that effects would have been detected if we utilized a shorter time frame between exposure and outcome. Nevertheless, a segment of the population continues to experience symptoms of poor mental health years after natural disaster exposure [32–36]. A recent multilingual systematic review found that specifically anxiety and depression symptoms remain elevated for years after exposure to disasters and pandemics and that young people having higher rates than adult populations (*p* < 0.005) [39]. It, therefore, merits understanding if patterns can be detected in later developmental stages.  In terms of outcome measures, anxiety and depression symptoms were not measured in earlier data collection. We adjusted for a measure of past subjective wellbeing, but subjective wellbeing does not completely overlap with anxiety and depression constructs. Although widely used, standardized scales do not capture culturally specific expressions of anxiety and depression. Poor mental health is commonly expressed as somatization in non-Western societies. For instance, the Luo of South Sudan define depression as “*nger yec*,” a conglomeration of symptoms that overlap with Western definitions of depression but also include stomach pain and diarrhea [71]. Similar somatic symptoms have been found among Ethiopian [72], Indian [73], Peruvian [74], and Vietnamese populations [75]. Last, these selected scales did not screen for other expressions of poor mental health. It may be that young adults had other mental health conditions, such as PTSD, which were outside of the purview of this study.” |
| Interpretation | 20 | Give a cautious overall interpretation of results considering objectives, limitations, multiplicity of analyses, results from similar studies, and other relevant evidence | 8-12 | “Young adults who lived in communities that were exposed to any natural hazard exposure pattern did not have substantially different GAD-7 and PHQ-8 scores from their unexposed peers. Prior exposure was not associated with increased risk of later anxiety and depression symptoms in young adulthood in these four countries, regardless of context, and effects were not magnified when young people were exposed at both time points. Past studies have found mixed associations between exposure to natural hazards and mental health, likely due to differences in exposure measurement, study population, and study design. A global meta-analysis contrasting disaster exposed to unexposed groups and incorporating pre-post testing found slight increases in depression among the exposed population (standardized mean difference [SMD] = 0.55, 95% CI: 0.05-1.06). Anxiety symptoms, however, did not conclusively differ [38]. Specific to children, Norris et al. [6] found mixed but generally worse mental health symptoms among younger age groups as compared to adults across multiple studies. Another meta-analytic study similarly estimated a slight increase in combined anxiety and depression metrics after exposure for youth ($r$ = 0.18, 95% CI: 0.14, 0.22) [21], and a recent systematic review of post-disaster mental health recovery from 29 countries found that depression remained elevated for years after natural disaster or pandemic exposure among young people and at a higher rate than in adult populations [39]. Amongst these studies, exposure was commonly measured in low- and middle-income countries as residence in a geographic area affected by the natural hazard (e.g., [25–30]). The current literature tends to indicate that exposure to natural hazards induces a slight but lasting increase in depression and anxiety symptoms among young people, with additional variation depending on the severity, duration, and personal impact of the natural hazard. In contrast, this study generally did not find evidence that exposure to natural hazards related to anxiety or depression symptoms. The findings support possible conclusions about the frequency and historical/longitudinal patterns of exposure (i.e., repeat exposure, exposure in adolescence/young adulthood, and exposures in early life) which are not typical of studies that examine one natural hazard event, and they complement existing evidence involving “dose” of exposure from proximity or severity to natural hazards.  The type and severity of traumatic experiences over one’s lifetime and during the natural hazard may be most predictive in determining who experiences poor mental health. Tang et al. [40] found that across studies of children, depression symptoms only significantly increased among subgroups that had prior experiences of trauma or specific negative experiences during and after the natural hazard (i.e., being trapped during the disaster; experiencing injury, fear, or bereavement during the disaster; witnessing injury or death; or having poor social support). Personal behaviors and worldviews additionally act to protect or exacerbate symptoms. Stoicism and maladaptive coping, such as venting and distraction, can lead to greater deterioration of mental health, whereas acceptance, positive reframing, and humor are protective [41]. Similarly, individuals that use religious coping to find meaning and positive lessons in negative events often have overall better mental health [42]. Young people, likewise, are embedded within families, communities, and societies that influence their mental health symptoms and recovery. Familial and social support as well as the overall sociopolitical context influence the development of symptoms of poor mental health after natural hazards [21,43,44]. This nuance collectively points to a complicated pattern of individual and socioecological factors that may modify the relationship between natural hazards and mental health. Although our study sought to adjust for all meaningful pre-exposure covariates in the dataset, individual, relational, and societal dimensions of mental health response are not fully understood or sufficiently captured in data on disaster-related mental health. Furthermore, a lack of consensus exists on how to best operationalize exposure, and measures of exposure at the community or individual level may differ in their sensitivity. Exposure has been constructed using individual disaster-related stressors or area-level measures of proximity, severity, and general residence in an affected area [24]. These measurements merit further exploration among young people in future research.  Unexpectedly, young women in Ethiopia exhibited lower GAD-7 scores (less reported anxiety) when living in communities that were repeatedly exposed to natural hazards at both time points. The literature largely finds that women have worse mental health symptomology than men when exposed to natural hazards [6,21–23]. Ethiopia is likely no exception. Of the limited evidence, a cross-sectional study from the capital of Addis Ababa found that woman had 1.74 (95% CI: 1.21, 2.50) higher adjusted odds of posttraumatic stress disorder (PTSD) after a landslide [45]. School-based samples among adolescents and young adults from various parts of the country further indicate that female gender is associated with higher depression and anxiety symptoms [46–48]. The lower anxiety symptoms among repeatedly exposed young women in Ethiopia may be an artifact of unmeasured confounding from factors that were not included in this study. One such covariate would be the structure of disaster relief aid. It is plausible that a greater amount of gender-sensitive aid was allocated to communities where natural hazards repeatedly occurred, as compared to communities that did not experience natural hazards. Ethiopia receives some of the world’s highest amount of foreign aid and hosts several national safety net programs that provide gender-responsive support to households affected by drought and other climate-related shocks [49,50]. It may be that young women in affected communities benefited from this investment in early childhood disproportionate to their peers and therefore, had lower anxiety for their future wellbeing when repeated natural hazards occurred. When baseline services are not well-distributed nationally, and one group receives targeted aid, asymmetries commonly occur [51]. Ethiopia has had mixed success in its aid distribution and gender-responsiveness [52,53]; however, qualitative evidence collected as part of a longitudinal study with Ethiopian adolescents and young adults, aged 10 to 20 years, corroborates that climate mitigation strategies led to investment in gender-sensitive social protection programs, which positively impacted young women and girls [50]. Young women who lived in communities where these programs were implemented may have benefited over the long-term. Furthermore, the study measured anxiety and depression during the height of the COVID-19 pandemic. Poor households in rural areas were 88.0% more likely to receive government assistance within the 10 months after the start of the pandemic than non-poor households. These households were disproportionately female headed and overlapped with areas of the country where natural hazards frequently occur [54]. COVID-19 investment could have lowered recent anxiety symptoms among young women in these communities. One’s perception of the government’s response to natural hazards may be as important as the actual aid received in influencing mental health symptoms. Although a different context, an estimated 18.0% of survivors of the Southeast Asian earthquake and tsunami in Thailand newly developed posttraumatic stress symptoms when they perceived that there was a low level of government support [55].” |
| Generalisability | 21 | Discuss the generalisability (external validity) of the study results | 12-13 | “The study employed psychometrically validated mental health scales that have been widely used with young adults in a variety of cultural settings [61–66]. Depression and general anxiety are the two most common mental health symptoms and are common after natural hazard exposure [40,67].” |
| Other information | | |  |  |
| Funding | 22 | Give the source of funding and the role of the funders for the present study and, if applicable, for the original study on which the present article is based | 34 | **“Funding**  This analysis was supported by the National Institute of Environmental Health Sciences of the National Institutes of Health under Award Number T32ES007322 and K25ES034064. The content is solely the responsibility of the authors and does not necessarily represent the official views of the National Institutes of Health.” |

# Supplementary Table S3. Natural hazard exposure by country and time point

| Ethiopia | | India | |
| --- | --- | --- | --- |
| Time point 1 | Time point 2 | Time point 1 | Time point 2 |
| Avalanche/ mud slide | - | - | - |
| - | - | - | Cyclone/tornado/hurricane |
| Drought | Drought | Drought | Drought |
| Earthquake | - | - | - |
| - | - | - | - |
| Flooding | Flooding | Flooding | Flooding |
| - | - | - | - |
| - | - | - | - |
| Other | Other | Other | Other |

| Peru | | Vietnam | |
| --- | --- | --- | --- |
| Time point 1 | Time point 2 | Time point 1 | Time point 2 |
| Avalanche/ mud slide | Avalanche/ mud slide | Avalanche/ mud slide | - |
| Cyclone/tornado/hurricane | Cyclone/tornado/hurricane | Cyclone/tornado/hurricane | Cyclone/tornado/hurricane |
| Drought | Drought | Drought | - |
| Earthquake | Earthquake | - | - |
| - | Erosion/cracks/landslide | - | - |
| Flooding | Flooding | Flooding | Flooding |
| Frost/cold front/hailstorm | Frost/cold front/hailstorm | - | Frost/cold front/hailstorm |
| - | - | - | Severe storm |
| Other | Other | Other | Other |

Responses were collected as binaries (yes/no) if event occurred or did not occur within a community at each time point. Non-geophysical shocks or climate-related events were excluded from analysis. Dashes indicate that a natural hazard type did not occur or were not collected at that time point.

# Supplementary Table S4. Targeted causal parameters for natural hazard exposure regimes

| Time point 1: *𝔼* ${[Y}_{1,0}$] *− 𝔼* ${[Y}_{0,0}$] |
| --- |
| Time point 2: *𝔼* ${[Y}_{0,1}$] *− 𝔼* ${[Y}_{0,0}$] |
| Both time points: *𝔼* ${[Y}_{1,1}$] *− 𝔼* ${[Y}_{0,0}$] |

$Y_{1,0}$,$Y_{0,1}$, and $Y_{1,1}$ denote potential outcomes for a child under natural hazard exposure regimes at time point 1, time point 2, and both time points. The subscripts indicate the time point of exposure and if a community was exposed or unexposed (0 = unexposed and 1 = exposed). $Y_{0,0}$ = potential outcome under an exposure history of never being exposed.

# Supplementary Table S5. List of covariates

| Individual-level | Household-level | Community-level |
| --- | --- | --- |
| Young adult’s biological sex | Size of household | Urban/rural^a,c,d^ |
| Young adult’s primary language^a,c,d^ | Own land^b^ | Population |
| Young adult’s religion | Own house^a,d^ | Theft/robbery^b,d^ |
| Young adult’s ethnic group/caste^c,d^ | Own animals^a,d^ | Violent crime^a,b^ |
| Young adult’s level of education^a^ | Food insecurity of household^b^ | Prostitution^a,b,c^ |
| Young adult almost died in infancy |  | Youth crime^a,b^ |
| Young adult ever drank alcohol^b,c,d^ |  | Social worker in community^c,d^ |
| Young adult ever smoked^a,b^ |  | Ever previous epidemic^b^ |
| Young adult’s recent subjective wellbeing score |  |  |
| Primary caregiver’s age |  |  |
| Primary caregiver’s relationship to the young adult^b,c,d^ |  |  |
| Primary caregiver ever received schooling |  |  |
| Primary caregiver recently lost employment^b,d^ |  |  |
| Parents divorced in childhood^b^ |  |  |
| Mother died in childhood^b,c,d^ |  |  |
| Mother ill in childhood |  |  |
| Father ill in childhood |  |  |

^a^ = not used in Ethiopia, ^b^ = not used in India, ^c^ = not used in Peru, ^d^ = not used in Vietnam.

Covariate categories:

1. Primary caregiver’s relationship to young adult is biological parent (yes/no) in Ethiopia.
2. Young adult’s religion is Islam (yes/no) or Christian Orthodox (yes/no) in Ethiopia; Hindu (yes/no) in India; has no religion (yes/no) in Peru; and Buddhist (yes/no) or minority religions (yes/no) in Vietnam.
3. Young adult’s primary language spoken is Telugu (yes/no) in India.
4. Young adult’s ethnic group is Amhara (yes/no) in Ethiopia; and castes are backwards caste (yes/no), scheduled caste (yes/no), or no caste (yes/no) in India.
5. Young adult’s highest level of education is primary (yes/no), secondary (yes/no), or university (yes/no) in India; received education (yes/no) in Peru; and received primary or secondary education (yes/no) in Vietnam.

# 
